# Expansion of Auxiliary Activity Family 5 sequence space via biochemical characterization of six new copper radical oxidases

**DOI:** 10.1128/aem.01014-24

**Published:** 2024-07-02

**Authors:** Jessica K. Fong, Yann Mathieu, Minh Tri Vo, Annie Bellemare, Adrian Tsang, Harry Brumer

**Affiliations:** 1 Michael Smith Laboratories, University of British Columbia, Vancouver, British Columbia, Canada; 2 Department of Chemistry, University of British Columbia, Vancouver, British Columbia, Canada; 3 Centre for Structural and Functional Genomics, Concordia University, Montreal, Quebec, Canada; 4 Department of Biochemistry and Molecular Biology, University of British Columbia, Vancouver, British Columbia, Canada; 5 Department of Botany, University of British Columbia, Vancouver, British Columbia, Canada; Kyoto University, Kyoto, Japan

**Keywords:** carbohydrate-active enzyme, Auxiliary Activity Family 5, copper radical oxidase, galactose oxidase, alcohol oxidase, HMF oxidase

## Abstract

Bacterial and fungal copper radical oxidases (CROs) from Auxiliary Activity Family 5 (AA5) are implicated in morphogenesis and pathogenesis. The unique catalytic properties of CROs also make these enzymes attractive biocatalysts for the transformation of small molecules and biopolymers. Despite a recent increase in the number of characterized AA5 members, especially from subfamily 2 (AA5_2), the catalytic diversity of the family as a whole remains underexplored. In the present study, phylogenetic analysis guided the selection of six AA5_2 members from diverse fungi for recombinant expression in *Komagataella pfaffii* (syn. *Pichia pastoris*) and biochemical characterization *in vitro*. Five of the targets displayed predominant galactose 6-oxidase activity (EC 1.1.3.9), and one was a broad-specificity aryl alcohol oxidase (EC 1.1.3.7) with maximum activity on the platform chemical 5-hydroxymethyl furfural (EC 1.1.3.47). Sequence alignment comparing previously characterized AA5_2 members to those from this study indicated various amino acid substitutions at active site positions implicated in the modulation of specificity.

**IMPORTANCE:**

Enzyme discovery and characterization underpin advances in microbial biology and the application of biocatalysts in industrial processes. On one hand, oxidative processes are central to fungal saprotrophy and pathogenesis. On the other hand, controlled oxidation of small molecules and (bio)polymers valorizes these compounds and introduces versatile functional groups for further modification. The biochemical characterization of six new copper radical oxidases further illuminates the catalytic diversity of these enzymes, which will inform future biological studies and biotechnological applications.

## INTRODUCTION

There is an increasing emphasis on the advancement of a circular (bio)economy, including the development of more sustainable materials and industrial processes, due to the negative environmental impacts that result from our overconsumption of non-renewable resources (1–5). One avenue to address this is to utilize enzymes as bio-based catalysts. Enzyme discovery continues to play an important role toward the development of efficient enzymes for more environmentally friendly industrial applications (6–8). In particular, oxidative reactions encompass several important chemical transformations utilized in the pharmaceutical, agricultural, and biofuel industries, to name a few. Replacement of chemical oxidants, many of which produce hazardous waste, with oxidase enzymes as industrial (bio)catalysts is particularly attractive due to the low-risk waste streams and milder reaction condition requirements associated with enzymes (9, 10).

In this context, copper radical oxidases (CROs), which comprise Auxiliary Activity Family 5 (AA5) within the carbohydrate-active enzymes classification (11, 12), have become attractive targets as biocatalysts (13, 14). CROs catalyze the two-electron oxidation of primary alcohols or aldehydes (gem-diols) to their corresponding aldehyde or carboxylic acid products, respectively, using O_2_ as a terminal electron acceptor, generating H_2_O_2_ as a co-product. Within the active site, CROs coordinate a mononuclear copper metal center, which is redox-coupled to a unique crosslinked tyrosyl-cysteinyl radical co-factor (15, 16). Fungal CROs constitute two subfamilies of AA5: Subfamily 1 (AA5_1), which contains glyoxal oxidases first discovered in 1987 from the white rot fungus, *Phanerochaete chrysosporium* (17), and Subfamily 2 (AA5_2), which includes the archetypal galactose-6-oxidase (*Fgr*GalOx) from the wheat blight pathogen, *Fusarium graminearum* (18, 19). There are also AA5 CROs, including those from bacteria and plants, that do not fall into these two originally defined subfamilies (11, 13).

Since its discovery in the 1960s, *Fgr*GalOx has been utilized for a wide range of biotechnological applications, such as the functional modification of galactose-containing polysaccharides for the development of functional materials (20–23), glycoprotein labeling (24–26), drug development (27, 28), production of flavor or fragrance compounds (29–31), etc.

Despite this decades-long history, enzymological research on AA5_2 has focused exclusively on *Fgr*GalOx and close *Fusarium* homologs. In 2015, two *Colletotrichum* AA5_2 enzymes (*Cgr*AlcOx and *Cgl*AlcOx) were discovered, which preferentially oxidized aliphatic alcohols over galactose-containing substrates (32). Since then, several other AA5_2 enzymes have been characterized, revealing a broad substrate specificity within the subfamily that can be categorized into three catalytic classes: traditional galactose-6-oxidases (GalOx, EC 1.1.3.12) (16), general alcohol oxidases (AlcOx, EC 1.1.3.7) (32), and aryl alcohol oxidases (AAO, EC 1.1.3.37) (33). Although a significant amount of CRO research has centered on their potential as biocatalysts, recent studies have also uncovered the biological importance of CROs in cell wall morphogenesis and as virulence factors in phytopathogenesis (34–39). Sustained interest in exploiting the diverse substrate scope of CROs for industrial biocatalysts, together with advancing our understanding of their biological roles, motivates further exploration of the AA5_2 sequence space.

**Fig 1:**
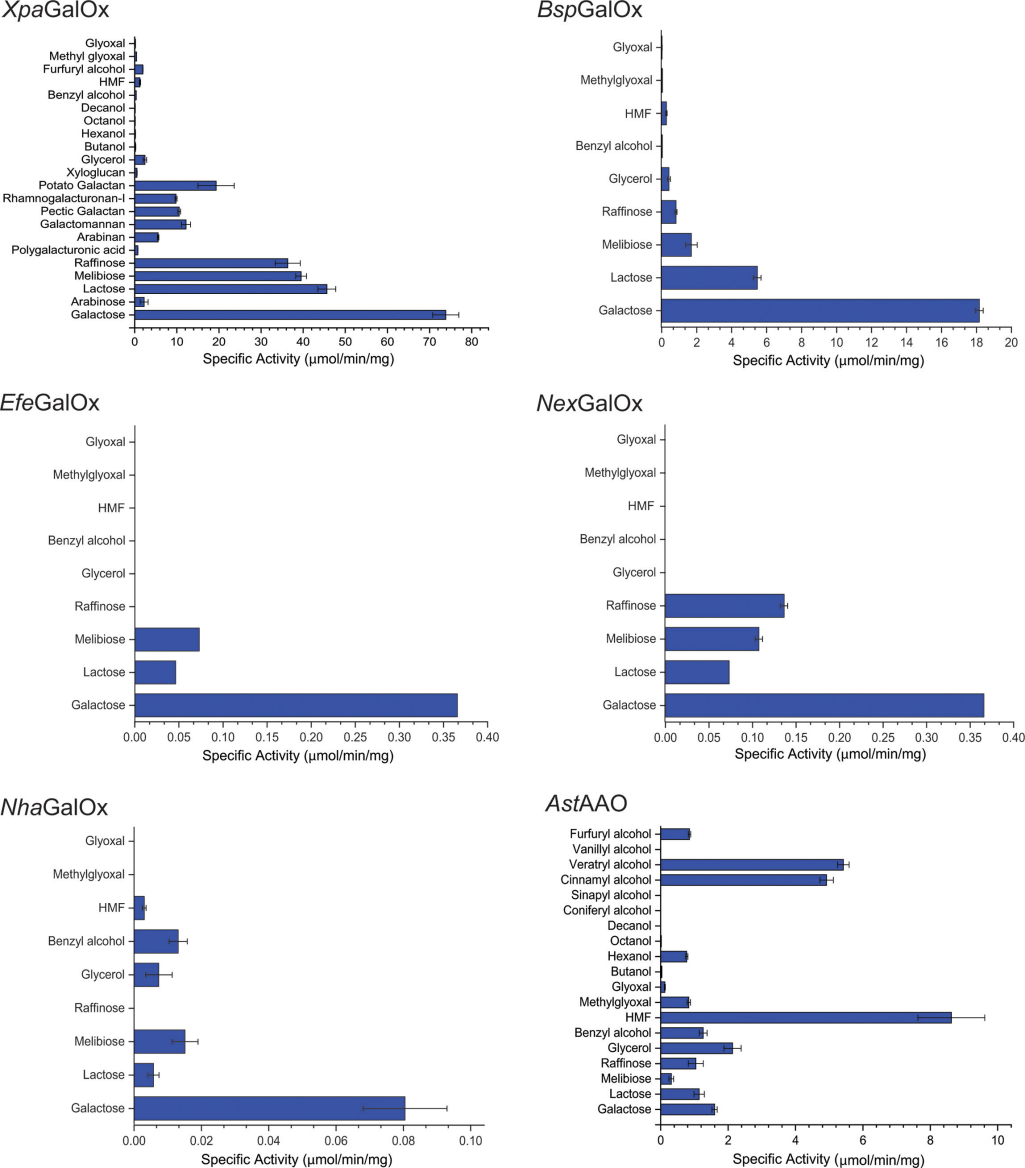
Specific activities of AA5_2 members on representative compounds. Individual values are presented in Table S2. Specific activity was determined using the HRP-ABTS-coupled assay with 50 mM sodium phosphate buffer, pH 7.0, at room temperature, except for *Nex*GalOx (50 mM sodium acetate buffer, pH 5.0). Measurements were performed in triplicate with 300 mM carbohydrates (galactose, lactose, melibiose, and raffinose), 300 mM glycerol, 10 mM aromatic alcohols [benzyl alcohol and 5-hydroxymethylfurfural (HMF)], and 10 mM aldehydes (methyl glyoxal and glyoxal). *Nha, Nectria haematococca; Efe, Epichloe festucae; Nex, Niesslia exilis; Ast, Acremonium strictum; Bsp, Bisporella* sp.; and *Xpa, Xanthoria parietina*.

Although the number of characterized AA5_2 members has significantly increased over the last few years [(32, 33, 40–44), reviewed in references (13, 14)], the subfamily remains largely underexplored (Fig. 1). To address this, phylogenetic analysis of AA5_2 sequences was performed to guide the selection of six new targets from several uncharacterized monophyletic groups for recombinant expression and subsequent biochemical characterization to reveal their activities.

## RESULTS

### Target selection and recombinant protein production

We presented previously a maximum likelihood (ML) phylogeny of 623 manually curated AA5_2 catalytic modules grouped into 38 clades, which was used to select targets for recombinant production and enzymatic characterization (40). Previously characterized AA5_2 members are broadly distributed around the tree (Fig. S1). Here, six AA5_2 targets were selected from distantly related (distal) and closely related (adjacent) clades to those of well-studied members to diversify the coverage of AA5_2. Targets chosen from the uncharacterized Clades 5 and 13 originate from *Bisporella* sp. *(Bsp*) and *Epichloe festucae (Efe*), both of which are plant endophytes, whereas the AA5_2 from the uncharacterized Clade 12 is from *Niesslia exilis* (*Nex*)*,* a saprophytic fungus. An AA5_2 from the lichen-forming fungus, *Xanthoria parietina (Xpa*), was chosen from Clade 3, which contains one characterized AA5_2 [*Mre*GalOx (40)]. Two AA5_2 targets from the fungi *Acremonium strictum (Ast*) and *Nectria haematococca (Nha*) were also chosen from Clade 14, which contains several well-characterized galactose and alcohol oxidases from different *Fusarium* species [*Fve*GalOx (45), *Fsu*GalOx (46), *Fox*AlcOx (40), *Fox*AAO, and *Fgr*AAO (41)].

All of the targets were successfully produced as secreted, hexahistidine-tagged proteins from *Komagataella pfaffii* (syn. *Pichia pastoris*) KM71H and purified via immobilized mobile affinity chromatography (Fig. S2). Typical protein yields using baffled shake-flasks ranged between 6 and 25 mg/L (Table S1). Treatment with PNGaseF under denaturing conditions resulted in changes in electrophoretic mobility corresponding to 3–19 kDa, indicating the presence of N-glycosylation on all of the purified AA5_2 enzymes (Fig. S2).

### Initial biochemical characterization

Activities of the recombinantly produced AA5_2 enzymes were screened with an initial panel of representative substrates known to be oxidized by AA5 members, i.e., galactose, lactose, melibiose, raffinose, glycerol, benzyl alcohol, 5-hydroxymethylfurfural (HMF), methyl glyoxal, and glyoxal. Based on previous observations with other AA5 members (33, 40, 41), screening assays were performed at room temperature and sodium phosphate buffer, pH 7.0. Most of the AA5_2 targets demonstrated canonical galactose-6-oxidase activity (EC 1.1.3.12) on galactose and galactosides. The exception was the AA5 member from *Acremonium strictum*, which was most active on HMF, indicative of general aryl alcohol oxidase (EC 1.1.3.37) activity (33, 40).

Galactose or HMF, as appropriate, was then used to determine the pH rate and temperature stability profiles for each CRO (Fig. S3 to S5). Generally, the enzyme targets displayed optimal activity between pH 6 and 8 in sodium phosphate buffer with approximately bell-shaped pH-rate profiles (Fig. S3), as observed for other characterized AA5_2 members (32, 33, 40–44). Exceptions were *Nha*GalOx, which displayed a sharp activity increase between pH 7.5 and 8.5, and *Nex*GalOx, which was most active in sodium acetate buffer at pH 5. In general, the AA5_2 targets were stable at 30°C, similar to previously characterized CROs and consistent with the mesophilic nature of their source fungi (Fig. S4 and S5). Interestingly, *Efe*GalOx retained full activity after 48 h of incubation at 50°C.

### Substrate specificities

Based on the specific activity values observed for each enzyme, additional substrates were screened. For *Xpa*GalOx, the most active galactose oxidase, this panel was expanded to include polysaccharides and alkanols, while for *Ast*AAO, additional aryl alcohols were assayed (Fig. 1; Table S2). A selection of the best substrates was subjected to detailed Michaelis-Menten kinetic analysis (Table 1; Fig. S6 to S9) at conditions (pH and temperature) based on the optima observed for each enzyme. These data are discussed for each CRO in the following sections.

**TABLE 1:** Michaelis-Menten kinetic parameters for selected substrates

Enzyme	Substrate	*K_M_* (mM)	*k*_cat_ (s^−1^)	*k*_cat_*/K_M_* (M^−1^ s^−1^)	pH	Temperature (°C)	Reference
*Fgr*GalOx	Galactose	82	503	6,370			(47)
		102	1,060	10,400			(48)
*Xpa*GalOx	Galactose	10 ± 0.2	241 ± 2	24,100	6.5	25	This work
	Lactose	58.4 ± 0.4	190 ± 2	3,250 (1,500)^*a*^			
	Melibiose	19.7 ± 2.8 (23.9 ± 5.5)^*b*^	206 ± 9 (234 ± 25)^*b*^	10,460 (9,790)^*b*^			
	Raffinose	6.9 ± 0.3	189 ± 4	27,400			
	Glycerol			(76)^*a*^			
	HMF			(330)^*a*^			
*Bsp*GalOx	Galactose	36.3 ± 1.4	52.1 ± 0.2	1,420	6.0	25	This work
	Lactose	186 ± 10	16.5 ± 0.6	89 (72)^*a*^			
	Melibiose	140 ± 7	13 ± 0.3	93 (54)^*a*^			
	Raffinose	156 ± 6	8.4 ± 0.2	54 (51)^*a*^			
	Glycerol			(3)^*a*^			
*Efe*GalOx	Galactose	8.2 ± 1.8 (8.1 ± 2.5)^*b*^	0.7 ± 0.1 (0.7 ± 0.1)^*b*^	85 (86)^*b*^	8.5	50	This work
*Nex*GalOx	Galactose	828 ± 78	7.5 ± 0.6	9 (7.2)^*a*^	5.0	30	This work
*Nha*GalOx	Galactose	930 ± 98	4.3 ± 0.4	5 (4)^*a*^	8.0	30	This work
*Ast*AAO	Galactose	931 ± 138	13.8 ± 1.3	15 (14)^*a*^	7.5	30	This work
	Lactose			(6)^*a*^			
	Melibiose	486 ± 68	2.1 ± 0.2	4 (3)^*a*^			
	Raffinose	75.3 ± 2.6	2.8 ± 0.1	37 (30)^*a*^			
	Glycerol	1,697 ± 163	30.6 ± 2.2	18 (18)^*a*^			
	Hexanol	2 ± 7.5	8.3 ± 0.7	130 (107)^*a*^			
	Benzyl alcohol			(230)^*a*^			
	HMF	2.0 ± 1.0 (9.4 ± 1.0)^*b*^	24.9 ± 1.4 (42.7 ± 2.2)^*b*^	12,400 (4,540)^*b*^			
	Cinnamyl alcohol	11.6 ± 1.0	43.8 ± 1.5	3,780 (4,800)^*a*^			
	Veratryl alcohol	1.8 ± 0.3 (6.4 ± 0.8)^*b*^	16.5 ± 1.5 (38.3 ± 2.6)^*b*^	9,160 (5,980)^*b*^			
	Furfuryl alcohol	24.0 ± 3.7	15.6 ± 1.7	650 (510)^*a*^			
	Methylglyoxal	45 ± 11	6.8 ± 1.2	150 (119)^*a*^			
	Glyoxal			(15)^*a*^			

^*a*^ Values in parentheses correspond to *k*_cat_/*K*_*M*_ values determined from the slopes of linear fits to initial-rate kinetic data substrate concentrations well below saturation (see Fig. S6 to S9); individual *K*_*M*_ and *k*_cat_ values were not calculated.

^*b*^ Kinetic parameters were obtained by fitting a modified Michaelis-Menten equation including a term for substrate inhibition.

#### *Xpa*GalOx

**Fig 2:**
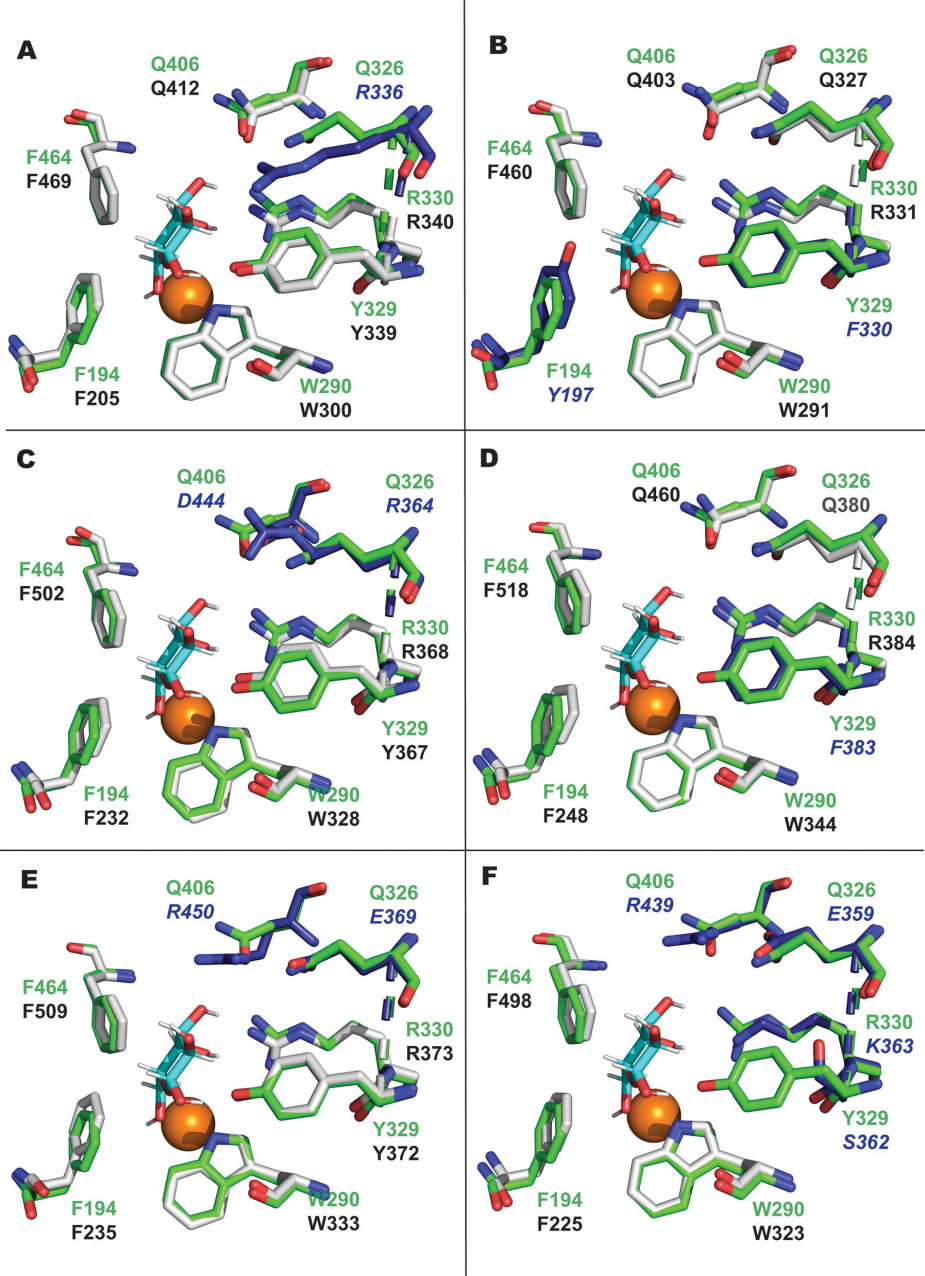
Tertiary structural comparison of AA5 CRO active sites. Structural models of (**A**) *Xpa*GalOx, (**B**) *Bsp*GalOx, (**C**) *Efe*GalOx, (**D**) *Nex*GalOx, (**E**) *Nha*GalOx, and (**F**) *Ast*AAO were generated with AlphaFold3 (73) and superposed with an experimental structure of *Fgr*GalOx [PDB 1gof (19), green amino acids], into which galactose was docked previously (40). Conserved residues within respective AA5_2 members from this work are shown as gray sticks with black labels, while non-conserved residues are displayed as blue sticks and labels. Galactose and copper are shown as cyan sticks and orange spheres, respectively. In all cases, AlphaFold did not generate the corresponding active-site Cys-Tyr crosslink (19), but modeled these as two independent sidechains; this did not affect the overall analysis.

The lichen-forming symbiotic fungus, *Xanthoria parietina (Xpa*), commonly associates with green algae and has found use as a biomonitor for the qualitative detection of environmental metal pollution due to its robust tolerance to heavy metals (49). *X. parietina* encodes an AA5_2 member, *Xpa*GalOx, which is in the divergent Clade 3 (Fig. 1) together with a recently characterized galactose oxidase [*Mre*GalOx (40)] with which it shares 59% sequence identity. Similarly, *Xpa*GalOx shares 58% identity with *Fgr*GalOx (19) (Table S1). Initial activity screens with a small panel of representative substrates showed that *Xpa*GalOx had the highest specific activity on galactose and was relatively good at oxidizing galacto-oligosaccharides such as lactose, melibiose, and raffinose. *Xpa*GalOx also exhibited low specific activities on glycerol, benzyl alcohol, and, interestingly, the classic AA5_1 substrate, methyl glyoxal (Fig. 2). Based on the high specific activities observed on galacto-oligosaccharides, the substrate panel was extended to more relevant plant-based carbohydrates. The activity was detected on arabinose, arabinan, galactomannan, galactan, and rhamnogalacturonan-I (Fig. 1). Initial rate kinetics showed that *Xpa*GalOx had comparable catalytic efficiencies for galactose and raffinose (Table 1). *Xpa*GalOx also showed good catalytic efficiency for melibiose. As listed in Table 2, *Xpa*GalOx displayed a *k*_cat_ value, which was two- and fourfold lower than the reported values for *Fgr*GalOx [503 s^−1^ (47) and 1,060 s^−1^ (48)]. Interestingly, *Xpa*GalOx showed a 10-fold decrease in the *K*_*M*_ for galactose, which resulted in a two- and fourfold increase in specificity compared to the archetype, *Fgr*GalOx, which has reported *k*_cat_/*K*_*M*_ values of 6,370 M^−1^ s^−1^ (47) and 10,400 M^−1^ s^−1^ (48), respectively.

**TABLE 2:** Comparison of amino acid substitutions in key active site positions of several characterized AA5_2 members and the archetype *Fgr*GalOx^*a*^

Enzyme	Organism	Active site amino acids							GenBank/JGI accession	Reference
		Radical stabilization	Active site shape/substrate recognition							
*Fgr*GalOx	*Fusarium graminearum*	**W290**	**F194**	**Q326**	**Y329**	**R330**	**Q406**	**F464**	AAO95371	(16, 19)
*Fsa*GalOx	*Fusarium sambucinum*	**W**	**F**	**Q**	**Y**	**R**	**Q**	**F**	AIR07394	(50)
*Fau*GalOx	*Fusarium austro- americanum*	**W**	**F**	**Q**	**Y**	**R**	**Q**	**F**	AAA16228	(45)
*Mre*GalOx	*Mytilinidion resinicola*	**W**	**F**	**Q**	*F*	**R**	**Q**	**F**	XP_033570565	(40)
*Exe*GalOx	*Exophiala xenobiotica*	**W**	**F**	**Q**	*F*	**R**	**Q**	**F**	KIW55415	(40)
*Fox*GalOxB	*Fusarium oxysporum*	**W**	*M*	**Q**	*F*	**R**	**Q**	**F**	FOTG_04629	(40)
*Pfe*GalOx	*Penicillium fellutanum*	**W**	*Y*	**Q**	**Y**	**R**	**Q**	**F**	jgi|Penfe1|382062	(40)
***Xpa* GalOx**	*Xanthoria parietina*	**W**	**F**	*R*	**Y**	**R**	**Q**	**F**	jgi|Xanpa2|1578647	This work
***Bsp* GalOx**	*Bisporella* spp.	**W**	*Y*	**Q**	*F*	**R**	**Q**	**F**	jgi|Bissp1|110239	This work
***Efe* GalOx**	*Epichloe festucae*	**W**	**F**	*R*	**Y**	**R**	*D*	**F**	ACN30267	This work
***Nex* GalOx**	*Niesslia exilis*	**W**	**F**	**Q**	*F*	**R**	**Q**	**F**	jgi|Nieex1|192818	This work
***Nha* GalOx**	*Nectria haematococca*	**W**	**F**	*E*	**Y**	**R**	*R*	**F**	XP_003039318	This work
*Fve*GalOx	*Fusarium verticillioides*	**W**	**F**	*E*	**Y**	*K*	**Q**	**F**	ADG08188	(45)
*Fsu*GalOx	*Fusarium subglutinans*	**W**	**F**	*E*	**Y**	*K*	**Q**	**F**	ADG08187	(46)
*Pru* AlcOx/GalOx	*Penicillium rubens*	**W**	*Y*	*D*	**Y**	**R**	*E*	**F**	CAP96757	(40, 43)
*Cgr*RafOx	*Colletotrichum graminicola*	*Y*	**F**	*A*	*W*	**R**	*S*	**F**	EFQ36699	(42)
*Cgr*AlcOx	*Colletotrichum graminicola*	*F*	*W*	*G*	*F*	*M*	*T*	**F**	EFQ30446	(32)
*Cg*lAlcOx	*Colletotrichum gloeosporioides*	*F*	*W*	*L*	*F*	*M*	*T*	**F**	jgi|Gloci1|1901294	(32, 40)
*Por*AlcOx	*Pyricularia oryzae*	*F*	**F**	*G*	*L*	*Y*	*T*	**F**	XP_003719369	(40)
*Asy*AlcOx	*Aspergillus sydowii*	*F*	*Y*	*H*	*D*	**R**	*V*	**F**	XP_040706357	(40)
*Afl*AlcOx	*Aspergillus flavus*	**W**	*Y*	*L*	**Y**	*H*	*E*	**F**	KAF7627372	(40)
*Fox*AlcOx	*Fusarium oxysporum*	**W**	**F**	*D*	*S*	*K*	*A*	**F**	EXL65576	(40)
*Fox*AAO	*Fusarium oxysporum*	**W**	**F**	*E*	**Y**	*K*	**Q**	**F**	XP_018246910	(41)
*Fgr*AAO	*Fusarium graminearum*	**W**	**F**	*E*	**Y**	*K*	**Q**	**F**	XP_011322138	(41)
*Cgr*AAO	*Colletotrichum graminicola*	*Y*	**F**	*E*	*W*	**R**	*T*	**F**	EFQ27661	(33)
***Ast* AAO**	*Acremonium strictum*	**W**	**F**	*E*	*S*	*K*	*R*	**F**	jgi|Acrst|1377707	This work

^*a*^ Conserved residues in relation to *Fgr*GalOx are indicated in bold font and non-conserved residues are indicated in italic font, based on primary and tertiary structural alignments. AA5_2 members characterized in this present study are indicated in underlined, bold font.

The product(s) following galactose oxidation by *Xpa*GalOx were assessed after the incubation of 300 mM of galactose with *Xpa*GalOx. The proton nuclear magnetic resonance (NMR) spectrum of the reaction showed that *Xpa*GalOx oxidized the C6-OH of galactose with 35% conversion to produce the corresponding aldehyde (Fig. S10), typical of galactose 6-oxidases (EC 1.1.3.9).

#### *Bsp*GalOx

*Bisporella* sp. is a widespread saprobic ascomycete, which is also frequently isolated as a plant endophyte (51). The protein encoded by *Bsp_*PMI857 (JGI accession) is located in Clade 5, which contains no other characterized members (Fig. S1). *Bsp*GalOx shares 55% sequence identity with *Fgr*GalOx (19) (Table S1). *Bsp*GalOx exhibited the highest specific activity on galactose and lower activities on galacto-oligosaccharides. Minimal activities were also observed on glycerol and HMF (Fig. 1). Although *Bsp*GalOx was most active on galactose and had a lower *K*_*M*_ value for galactose, it demonstrated a 5- and 10-fold lower catalytic efficiency for galactose compared to the reported values of 6,370 M^−1^ s^−1^ (47) and 10,400 M^−1^ s^-1^ (48), respectively, for *Fgr*GalOx (Table 1). Catalytic efficiencies of *Bsp*GalOx for lactose, melibiose, and raffinose were 15, 15, and 25 times lower, respectively, compared to galactose (Table 1).

#### *Efe*GalOx

*Epichloe festucae* is an endophytic fungus associated with cool-season grasses, often conferring insect resistance to grass hosts due to the production of toxic alkaloids (52). *Efe*GalOx is located alone in Clade 13 and shares 65% sequence identity with *Fgr*GalOx (19) (Fig. S1; Table S1). The adjacent Clade 14 contains several characterized members from various *Fusarium* species [*Fve*GalOx (45), *Fsu*GalOx (46), *Fox*AlcOx (40), *Fox*AAO, and *Fgr*AAO (41)]. Initial activity screens showed that galactose was the best substrate for *Efe*GalOx, and minimal activity was observed on lactose and melibiose. No activity was detected on raffinose, glycerol, benzyl alcohol, and HMF nor was there any activity on the classic AA5_1 substrates, methyl glyoxal and glyoxal (Fig. 1). Although the highest specific activity was observed on galactose, initial rate kinetics showed that *Efe*GalOx had a 120-fold lower catalytic efficiency for galactose, compared to *Fgr*GalOx (Table 1). Notably, *Efe*GalOx had a *K*_*M*_ value for galactose that was 10-fold lower than that of *Fgr*GalOx. Despite this, a significantly lowered *k*_cat_ resulted in a ca. 100-fold lower *k*_cat_/*K*_*M*_ value compared to *Fgr*GalOx (Table 2).

#### *Nex*GalOx

The fungus *Niesslia exilis* is a saprotrophic filamentous fungus that inhabits decaying plant matter such as leaf litter or woody debris (53). *Nex*GalOx is in Clade 12, which contains no other characterized members, and is adjacent to Clade 13, which contains the aforementioned *Efe*GalOx (Fig. S1). *Nex*GalOx shares 60% and 65% sequence identity with *Efe*GalOx and *Fgr*GalOx (19), respectively (Table S1). Low specific activities were measured on galactose and galacto-oligosaccharides (lactose, melibiose, and raffinose), and no activity was observed on glycerol, benzyl alcohol, HMF, methyl glyoxal, and glyoxal, similar to the substrate profile of *Efe*GalOx (Fig. 1). A higher *K*_*M*_ value and lower *k*_cat_ value resulted in catalytic efficiencies for galactose that were over 700-fold lower compared to *Fgr*GalOx (Table 1).

#### *Nha*GalOx

*Nectria haematococca (*asexual form: *Fusarium solani*) is one of over 50 members of the *Fusarium solani* species complex (54). Members of this complex are known to cause disease in over 100 genera of plants. *Nha*GalOx is located in Clade 14, which also contains the *Fusarium* CROs *Fve*GalOx (45), *Fsu*GalOx (46), *Fox*AAO, *Fgr*AAO (41), and *Fox*AlcOx (40), as well as *Ast*AAO discussed below (Fig. S1). *Nha*GalOx shares 71% sequence identity with *Fgr*GalOx (19). Similarly, *Nha*GalOx also shares 70% identity with *Fox*AAO, *Fgr*AAO, and *Fox*AlcOx from the same clade (Fig. S1). The highest specific activity was measured on galactose, with lower levels of activity detected on lactose, melibiose, glycerol, benzyl alcohol, and HMF; no activity was detected on methyl glyoxal and glyoxal (Fig. 1). Despite the low specific activities measured, the broad substrate profile of *Nha*GalOx validates the grouping of *Fusarium* AA5_2 homologs in Clade 14. Although *Nha*GalOx was most active on galactose, initial rate kinetics show that a combination of higher *K*_*M*_ and lower *k*_cat_ values (Table 1) resulted in a catalytic efficiency on galactose that was over 1,000 times lower than that of *Fgr*GalOx.

#### *Ast*AAO

*Acremonium strictum* is an environmentally widespread soil-dwelling saprotroph that has also been shown to be involved in a range of plant endophytic and parasitic relationships. *A. strictum* has also been reported to be an opportunistic human pathogen, infecting immunocompromised patients (55, 56). *Ast*AAO is in Clade 14 together with the aforementioned *Nha*GalOx and several characterized *Fusarium* CROs (Fig. S1). *Ast*AAO shares 65% and 69% sequence identity with *Fgr*GalOx (19) and *Nha*GalOx, respectively. Comparable to *Nha*GalOx, *Ast*AAO shares 72% sequence identity with *Fox*AAO, *Fgr*AAO (41), and *Fox*AlcOx (40) (Table S1). Initial activity screens showed that *Ast*AAO was most active on HMF and had lower specific activities on carbohydrates. As such, the substrate panel was extended to include other aliphatic and aromatic alcohols (Fig. 1). Activity was detected on cinnamyl alcohol, veratryl alcohol, and furfuryl alcohol. Interestingly, activity was detected on hexanol but not on butanol, octanol, and decanol. Activity was also detected on methylglyoxal and glyoxal, which are typical substrates of AA5_1 CROs (Fig. 1). Initial rate kinetics showed that *Ast*AAO has comparable catalytic efficiencies on HMF, cinnamyl alcohol, and veratryl alcohol (Table 1). Substrate inhibition was also observed for several substrates, namely HMF and veratryl alcohol (Fig. S9). In comparison with characterized AA5_2 CROs, *Ast*AAO showed a similar substrate profile to that of *Cgr*AAO (33) from *Colletotrichum graminicola,* despite sharing only 46% sequence identity (Table S1). Like *Cgr*AAO, the catalytic efficiency of *Ast*AAO for galactose was 700-fold lower than the highest reported *k*_cat_/*K*_*M*_ value of 10,400 M^−1^ s^−1^ for *Fgr*GalOx (48). Although HMF was one of the best substrates for *Ast*AAO, its catalytic efficiency was four times lower than that of *Cgr*AAO for HMF. Interestingly, despite being several fold less efficient on many *Cgr*AAO substrates, *Ast*AAO had a comparable catalytic efficiency for veratryl alcohol.

HMF is an important biomass-derived platform chemical, which can yield several useful chemical building blocks through its partial or complete oxidation. These include 2,5-dimethylfuran (DFF), 5-hydroxy-2-furancarboxylic acid, 5-formyl-2-furancarboxylic acid (FFCA), and 2,5-furandicarboxylic acid (FDCA) (57, 58). Individual AA5_2 CROs have been reported to oxidize HMF to varying degrees. For instance, *Cgr*AAO (33), *Fgr*AAO (41), and *Cgr*AlcOx (32) oxidize HMF to DFF, a mixture of DFF/FFCA and a mixture of FFCA/FDCA, respectively. Proton NMR analysis indicated that *Ast*AAO oxidized HMF to DFF and FFCA in the ratio of 60:40 (Fig. S11).

## DISCUSSION

Enzyme discovery plays an important role in advancing green chemistry and the development of a sustainable bioeconomy (59, 60). Oxidation chemistry constitutes ca. 30% of industrial chemical transformations (10); thus, the discovery and characterization of diverse CROs can stimulate the development of new oxidative biocatalysts. Indeed, an engineered CRO was central to the recent industrial development of a biocatalytic pathway to produce a key antiviral drug (61), and efforts continue to be focused on optimizing CRO catalysis and expanding CRO substrate specificities (28, 62, 63); see also reference (13). In parallel, as CROs are implicated in fungal morphogenesis and phytopathogenesis (34, 35), understanding CRO biochemistry, therefore, underpins understanding their roles in biology and potential as targets in the development of strategies to combat plant disease.

In the present study, six new CROs from a range of AA5_2 phylogenetic clades were biochemically characterized. Five were demonstrated to be canonical galactose-6-oxidases (*Xpa*GalOx, *Bsp*GalOx, *Efe*GalOx, *Nex*GalOx, and *Nha*GalOx) and one to be an aryl alcohol oxidase (*Ast*AAO). As shown previously by our group (40) and elaborated here, AA5_2 sequences separate into 38 well-supported clades with high bootstrap values (Fig. S1). However, the inclusion of the newly characterized members in this phylogeny further illustrates that galactose-specific (EC 1.1.3.12) and general alcohol oxidase activities (EC 1.1.3.7, EC 1.1.3.37, and EC 1.1.3.47) are dispersed throughout numerous clades and are not monophyletic. This likely reflects the high overall sequence identity and similarity observed in the family as a whole (Table S1), which confounds functional prediction.

No substrate or product complexes of AA5 members have been solved, so direct information on active-site interactions is currently lacking (19, 32, 33, 37, 64). However, site-directed mutagenesis and *in silico* studies have implicated several key active site residues in the modulation of substrate specificity (47, 65–67). Yet, these studies also indicate that substrate specificity cannot be readily attributed to the effects of any specific amino acids in a predictable manner. Substrate specificity may instead be due to a complex interplay between several factors, such as steric factors and hydrogen-bonding capabilities of amino acid side chains constituting the active site. To place the CROs from the present study in the context of previously characterized AA5_2 members, Table 2 and Fig. 2 present a comparison of key active site residues first established in *Fgr*GalOx.

Early work has established the roles that tryptophan (W290 in *Fgr*GalOx) in the second coordination sphere plays in the stabilization of the tyrosyl-cysteinyl radical cofactor and binding of galactose (47). More recently, mutagenesis of the corresponding tyrosine in the *Colletotrichum* aryl alcohol oxidase, *Cgr*AAO, to tryptophan (Y334W) greatly reduced activity on all aryl alcohols and greatly boosted activity on galactose-containing carbohydrates (33). The galactose 6-oxidases characterized in this work all contained a tryptophan in the position corresponding to W290 in *Fgr*GalOx. Interestingly, *Ast*AAO also possessed a tryptophan in this position, despite primarily oxidizing aryl alcohols (Table 2; Fig. 2F).

Other key residues include Y329 and R330 in *Fgr*GalOx (Table 2; Fig. 2), which are predicted to bind the C1-OH and C3-/C4-OH of galactose (40, 48, 66). The position corresponding to R330 in *Fgr*GalOx appears to be a strong modulator of substrate preference: the majority of characterized galactose oxidases retain this arginine (Table 2). Yet, some alcohol oxidases, i.e., *Cgr*AAO (33) and *Asy*AlcOx (40), also retain this arginine but substitute the second-shell tryptophan with tyrosine and phenylalanine, respectively. Also notable is that *Pru*AlcOx retains the corresponding tryptophan/arginine pair and has similar activity on galactose and other alcohols (40). This suggests that the R330 position in *Fgr*GalOx is necessary but not sufficient for determining galactose activity. Indeed, the growing body of data suggests that the tryptophan/arginine pair, corresponding to W290 and R330 in *Fgr*GalOx, are the key indicators of galactose 6-oxidase activity although the modulation of activity levels may involve more complex factors. For example, *Nex*GalOx and *Nha*GalOx, which have the W290/R330 pair, display galactose oxidase activity but are significantly less efficient at turning-over galactose when compared to *Fgr*GalOx.

F194 and F464 in *Fgr*GalOx (Table 2; Fig. 2) are proposed to provide a hydrophobic surface for interactions with galactose. Molecular modeling and site-directed mutagenesis suggest that F464, in particular, forms aromatic stacking interactions with the galactose ring (48). Interestingly, F464 (in *Fgr*GalOx) is conserved in all characterized CROs and is therefore not an indicator of GalOx, AlcOx, or AAO activity (Table 2). The position corresponding to F194 in *Fgr*GalOx, which is variably substituted with other aromatic residues among the characterized CROs (Tyr and Trp; Met in *Fox*GalOxB is anomalous), also does not correlate with substrate scope (Table 2).

Regarding the position corresponding to Y329 in *Fgr*GalOx, this residue is variably substituted by phenylalanine in other GalOxs [*Exe*GalOx, *Mre*GalOx, and *Fox*GalOxB (40), and *Bsp*GalOx and *Nex*GalOx (this work)]. All the aforementioned galactose oxidases had lower catalytic efficiencies for galactose compared to *Fgr*GalOx, which suggests that substitution of tyrosine to phenylalanine may affect overall levels of activity rather than modulate substrate preference. Interestingly, some alcohol oxidases, e.g., *Cgr*AlcOx (32) and *Cgr*AAO (33), also have aromatic substitutions at this position, while others [*Fox*AlcOx, *Por*AlOx, and *Asy*AlcOx (40)] contain non-aromatic residues. For example, *Ast*AAO from the present study has a serine in place of the tyrosine.

The glutamine residues in positions Q326 and Q406 in *Fgr*GalOx are also noteworthy (66–68). Loss of one or both glutamines is generally, but not exclusively (e.g., *Efe*GalOx), correlated with a loss of GalOx activity. A recent study on the engineering of *Cgr*AlcOx has shown that these glutamines increase affinity for galactose, likely through hydrogen bonding (65). Interestingly, *Xpa*GalOx, which has a twofold higher catalytic efficiency and 10-fold lower *K*_*M*_ on galactose compared to *Fgr*GalOx (Table 1), contains an arginine at the position corresponding to Q326 in *Fgr*GalOx (Table 2; Fig. 2A). Two other galactose oxidases from our present study (*Efe*GalOx and *Nha*GalOx) with over 100-fold lower catalytic efficiency on galactose contained Arg and Glu substitutions, respectively, at the position corresponding to Q326 in *Fgr*GalOx. Like *Xpa*GalOx, *Efe*GalOx (Table 2; Fig. 2C) also contains an Arg substitution at the Q326 (*Fgr*GalOx) position and has a *K*_*M*_ value for galactose that is 10 times lower than *Fgr*GalOx. The longer Arg residue could potentially extend further into the binding site to form additional hydrogen bonds that may improve galactose binding. *Efe*GalOx and *Nha*GalOx also contained Asp and Arg substitutions, respectively, at position Q406 in *Fgr*GalOx.

### Conclusion

The characterization of six new fungal AA5_2 orthologs in this study contributes comparative biochemical data that helps to refine the understanding and prediction of the catalytic potential of CROs. On one hand, the expansion of characterized CROs provides valuable information to guide enzyme selection for biocatalytic applications and further protein engineering efforts. On the other hand, this *in vitro* structure-function data can inform the identification of the true physiological substrates of CROs *in vivo*, which are generally unknown but integral to microbiology and phytopathogenesis.

## MATERIALS AND METHODS

### Chemicals and enzymes

All substrate stocks and buffer solutions were prepared with Ultrapure water (18.2 Ω) unless otherwise stated. Lyophilized powders of catalase from bovine liver (2,000–5,000 units/mg, Sigma) and horseradish peroxidase (Rz > 3.0, BioBasic Canada, Inc.) were used as received. All substrates and reagents were purchased from commercial sources (Sigma-Aldrich, VWR, or Fisher) and used without further purification.

### Sequence analysis and bioinformatics

A previous ML phylogenetic tree comprising 623 AA5_2 catalytic modules omitting any accessory modules (40) was used to select six uncharacterized AA5_2 sequences. AlphaFold 3 (69) was used to generate structural homology models of the target AA5_2 CROs with a copper ion, using the AlphaFold Server at https://golgi.sandbox.google.com/.

### DNA cloning and recombinant strain production

cDNA encoding the mature AA5_2 proteins, including any accessory modules, but without signal peptides, was commercially synthesized (Genewiz) and cloned directly into pPICZαA or pPICZαC using the EcoRI, ClaI, and XbaI restriction sites in flush with the sequence encoding the *Saccharomyces cerevisiae* α-factor signal peptide. The resulting constructs were transformed into chemically competent *Escherichia coli* DH5α by heat shock. Recombinant strains were produced as described in the Invitrogen EasySelect Pichia system manuals (Invitrogen). Briefly, 5 µg of recombinant plasmids containing target sequences were linearized with PmeI and transformed into *Komagataella pfaffii* (syn. *Pichia pastoris*) *KM71H* via electroporation. Transformed *K. pastoris* were spread on YPD agar plates containing 100 or 500 µg mL^−1^ of Zeocin. Zeocin-resistant transformants were isolated from plates.

### Large-scale protein production and purification

Large-scale protein production and purification were performed as previously described (40). Briefly, single colonies of *K. pastoris KM71H*-expressing clones were streaked onto agar plates containing Zeocin (100 or 500 µg/mL) and grown in the dark for 2 days (30°C). Precultures of 5 mL YPD and 0.5 mg/mL Zeocin were inoculated with a single colony and shaken for 30°C at 250 rpm for 8 h, after which 1 L of BMGY media in 4 L baffled flasks was inoculated with the precultures and left to grow overnight at 30°C, 250 rpm. Once the BMGY cultures reached an OD_600_ of 6–12, the cells were harvested by centrifugation (20 min, 20°C, 4,000 rpm) and resuspended using 400 mL of BMMY media, supplemented with 0.5 mM CuSO_4_ (70) and 3% methanol (vol/vol), then transferred into 2 L baffled flasks and shaken at 250 rpm at 16°C for 3 days. The cultures were fed 1% (vol/vol) methanol every 24 h, and on day 3, secreted proteins were separated from cells by centrifugation at 4,500 rpm for 20 min at 4°C. The supernatant was decanted, and the pH was adjusted to 7.4–8.0 by dropwise additions of 5 M NaOH. The resulting liquid was filtered through a 0.45 µm membrane and allowed to equilibrate for at least 12 h at 4°C.

The supernatant was passed through a 5 mL pre-packed Ni-NTA column, pre-equilibrated with 20 mM sodium phosphate buffer at pH 7.4 with 500 mM NaCl and 10 mM imidazole at 5 mL/min. Proteins were eluted with a linear gradient of 0%–100% of 500 mM imidazole in a 20 mM sodium phosphate buffer with 500 mM NaCl at 5 mL/min. The total elution volume was collected in 1 mL fractions, and fractions of interest were pooled and concentrated using a 10,000 MWCO Vivaspin centrifugal concentrator. Concentrated protein fractions (maximum 2 mL) were loaded onto a Superdex 75 size exclusion column pre-equilibrated with 200 mL of 100 mM sodium phosphate buffer at pH 7.0. Total elution volume was collected as 1.5 mL fractions, and fractions of interest were pooled and concentrated as mentioned previously. The proteins were aliquoted, flash frozen in liquid nitrogen, and stored at −70°C. SDS-PAGE was carried out using pre-cast 4%–20% (wt/vol) polyacrylamide gels in the presence of 2% (wt/vol) SDS under reducing conditions. Proteins were visualized using Coomassie blue R-250. Protein concentrations were determined by measuring *A*_280_ using extinction coefficients calculated with the ProtParam tool on the ExPASy server.

### Analytical protein deglycosylation

The presence of protein N-glycosylation was assessed by treatment with N-glycosidase F from *Flavobacterium meningosepticum* (PNGaseF, New England Biolabs) according to the manufacturer’s protocol. Briefly, deglycosylation experiments were performed under denaturing conditions by adding 5 µg pf protein to 10× Glycoprotein Denaturing Buffer and heated for 10 min at 100°C. The samples were subsequently diluted to 20 µL with GlycoBuffer 2 and tergitol-type NP-40 detergent. The samples were incubated for 1 h at 37°C after the addition of 1 µL of PNGaseF. Changes in protein mobility were assessed by SDS-PAGE stained and visualized by staining with Coomassie blue R-250. Molecular weights of proteins were estimated using a standard curve of the log (MW) versus Rf of the protein ladder (BLUelf).

### Substrate screen

The activity of target enzymes was surveyed on a variety of substrates using the HRP-ABTS-coupled assay in a reaction volume of 200 µL of 50 mM sodium phosphate buffer, pH 7.0, 0.25 mg/mL ABTS, and 0.1 mg/mL HRP at room temperature in a 96-well plate. Absorbance was measured at 415 nm on the BioTek Epoch microplate spectrophotometer. Galactose, lactose, melibiose, raffinose, glycerol, benzyl alcohol, hydroxymethylfurfural, methylglyoxal, and glyoxal were screened as representative carbohydrates (300 mM), polyol (300 mM), aryl alcohol, and aldehyde (10 mM) substrates.

### pH activity profile

Enzyme activity across a wide range of pH values was determined as described (40) using citrate-phosphate (pH 4.0–6.0), sodium phosphate (pH 6.0–8.5), glycine-NaOH (pH 8.0–9.5), and CHES (pH 9.0–10.5) buffers. Enzyme activity was measured using the HRP-ABTS-coupled assay in a 96-well plate format at room temperature (ca. 20°C) in 50 mM buffer with the best substrate as determined by the substrate screen (Fig. 1).

### Temperature stability profile

Temperature stability profiles of the target enzymes were determined by first diluting the stock protein in 50 mM sodium phosphate buffer at the previously determined pH optimum. The diluted protein was then pre-incubated in a thermocycler set to maintain a temperature gradient between 30°C and 50°C. Samples were taken out at different time intervals and enzyme activity was measured using the HRP-ABTS-coupled assay in a 96-well plate assay at room temperature. The best substrate determined from the substrate screen (Fig. 1) was used to assay enzyme activity.

### Michaelis-Menten kinetics

To determine the Michaelis-Menten parameters of the target enzymes, a range of substrate concentrations were used for each substrate. The reactions were performed using the coupled HRP-ABTS assay with 0.46 mM ABTS, 21 U/mL of HRP in 50 mM sodium phosphate buffer at pH between 6 and 8.5 and temperatures between 20°C and 30°C depending on pH profiles and temperature stability assays (except *Nex*GalOx, which was assayed in 50 mM sodium acetate, pH 5.0). Measurements were made in 1 mL plastic cuvettes using the Cary 60 UV-VIS spectrometer. The Michaelis-Menten equation was fit to the data using OriginPro software (OriginLab 9.85)

### Enzyme product analysis

HRP and catalase (1 mg/mL) were combined with 300 mM of galactose or 20 mM HMF in 50 mM sodium phosphate, pH 6.5, to a final volume of 1 mL. The reactions were initiated by adding 50 or 100 µg of purified *Xpa*GalOx and *Ast*AAO, respectively. For galactose oxidation, 0.1 mg/mL BSA was also added. The reactions were stirred at 400 rpm at room temperature for 24 h followed by enzyme removal through ultrafiltration (5 kDa cut-off Vivaspin column). Negative controls without additions of *Xpa*GalOx or *As*tAAO were prepared. Filtrate from the galactose oxidation was lyophilized after which the dried powder was dissolved in D_2_O. D_2_O was added to the filtrate from the HMF oxidation to a final composition of 10% (vol/vol). NMR experiments were performed on a Bruker AVANCE 400 Hz spectrometer. Peaks were identified by comparison with reference spectra (33, 40, 71). Percentage conversion was calculated from integration values of relevant peak areas.

## Data Availability

All data generated and analyzed during this study are included in this article and the associated supplementary information files. All nucleotide sequences, protein sequences, and protein structural information used in this work were extracted from public databases, i.e., GenBank (www.ncbi.nlm.nih.gov/genbank/), The Protein Data Bank (www.rcsb.org/), The CAZy database (www.cazy.org/), and JGI Mycocosm (mycocosm.jgi.doe.gov/mycocosm/home).

## SUPPLEMENTAL MATERIAL

Supplemental material aem.01014-24-s0001.pdf Tables S1 to S3; Figures S1 to S11.

ASM does not own the copyrights to Supplemental Material that may be linked to, or accessed through, an article. The authors have granted ASM a non-exclusive, world-wide license to publish the Supplemental Material files. Please contact the corresponding author directly for reuse.
