# Supplementary material for: Expansion of Auxiliary Activity Family 5 sequence space via biochemical characterization of six new copper radical oxidases
Source: Appl Environ Microbiol. 2024 Jul 2;90(7):e01014-24. doi: 10.1128/aem.01014-24 (PMC11267884; doi:10.1128/aem.01014-24)
Supplement: Supplemental material — Tables S1 to S3; Figures S1 to S11. [file aem.01014-24-s0001.pdf]

Expansion of Auxiliary Activity Family 5 sequence space via biochemical characterization of six new copper radical oxidases

Jessica K. Fong, Yann Mathieu, Minh Tri Vo, Annie Bellemare, Adrian Tsang, Harry Brumer

Supplementary Information

Supplementary Tables

Table S1. Purified protein yields of expressed AA5_2 enzymes from 400 mL of BMMY shake-flask culture after three days of methanol feeding.

Enzyme	Yield (mg)	Yield (mg/L)
*Xpa*GalOx	2.4	6
*Bsp*GalOx	5.5	13.8
*Efe*GalOx	3.7	9.3
*Nex*GalOx	10.1	25.3
*Nha*GalOx	4.7	11.8
*Ast*AAO	5.7	14.3

Table S2. Specific activities of AA5_2 members.*

	Enzymes and Specific Activities (µmol/min/mg enzyme)					
**Substrates**	***Xpa*GalOx**	***Bsp*GalOx**	***Efe*GalOx**	***Nex*GalOx**	***Nha*GalOx**	***Ast*AAO**
Galactose	74 ± 3	18 ± 0.2	(4.0 ± 0.4) x 10^{-1}	1.1 ± 0.1	(8.0 ± 1.0) x 10^{-2}	1.6 ± 0.1
Lactose	46 ± 2	5.5 ± 0.2	(5.0 ± 0.1) x 10^{-2}	(18 ± 0.2) x 10^{-2}	(0.6 ± 0.1) x 10^{-2}	1.1 ± 0.2
Melibiose	40 ± 1	1.7 ± 0.3	(7.0 ± 0.2) x 10^{-2}	(3.1 ± 0.2) x 10^{-1}	(2.0 ± 0.4) x 10^{-2}	0.3 ± 0.1
Raffinose	36 ± 1	0.8 ± 0.1	n.d.	(3.5 ± 0.4) x 10^{-1}	n.d.	1.0 ± 0.2
Glycerol	2.4 ± 0.4	0.4 ± 0.1	n.d.	n.d.	(0.7 ± 0.4) x 10^{-2}	2.1 ± 0.3
Benzyl alcohol	(4.0 ± 0.1) x 10^{-1}	(4.0 ± 0.2) x 10^{-2}	n.d.	n.d.	(1.3 ± 0.3) x 10^{-2}	1.3 ± 0.1
HMF	1.2 ± 0.2	(3.0 ± 0.4) x 10^{-1}	n.d.	n.d.	(3.0 ± 0.5) x 10^{-3}	8.7 ± 0.9
Methylglyoxal	(4.0 ± 0.1) x 10^{-1}	(0.7 ± 0.1) x 10^{-2}	n.d.	n.d.	n.d.	0.8 ± 0.1
Glyoxal	(1.4 ± 0.4) x 10^{-1}	(2.0 ± 0.4) x 10^{-2}	n.d.	n.d.	n.d.	(1.0 ± 0.1) x 10^{-1}
Arabinose	2.2 ± 0.9	n.t.	n.t.	n.t.	n.t.	n.t.
Polygalact-uronic acid	(8.0 ± 0.2) x 10^{-1}	n.t.	n.t.	n.t.	n.t.	n.t.
Arabinan	5.6 ± 0.2	n.t.	n.t.	n.t.	n.t.	n.t.
Galactomannan	12 ± 1	n.t.	n.t.	n.t.	n.t.	n.t.
Pectic galactan	11 ± 0.3	n.t.	n.t.	n.t.	n.t.	n.t.
Rhamnogalac-turonan I	9.8 ± 0.2	n.t.	n.t.	n.t.	n.t.	n.t.
Potato galactan	19 ± 4	n.t.	n.t.	n.t.	n.t.	n.t.
Xyloglucan	0.5 ± 0.1	n.t.	n.t.	n.t.	n.t.	n.t.

Butanol	(1.9 ± 0.7) x 10^{-1}	n.t.	n.t.	n.t.	n.t.	(3.0 ± 0.2) x 10^{-2}
Hexanol	(1.7 ± 0.3) x 10^{-1}	n.t.	n.t.	n.t.	n.t.	(7.7 ± 0.4) x 10^{-1}
Octanol	(13 ± 0.2) x 10^{-2}	n.t.	n.t.	n.t.	n.t.	(1.0 ± 0.2) x 10^{-2}
Decanol	(13 ± 0.3) x 10^{-2}	n.t.	n.t.	n.t.	n.t.	n.d.
Furfuryl alcohol	(20 ± 0.2) x 10^{-1}	n.t.	n.t.	n.t.	n.t.	(9.0 ± 0.4) x 10^{-1}
Coniferyl alcohol	n.t.	n.t.	n.t.	n.t.	n.t.	n.d.
Sinapyl alcohol	n.t.	n.t.	n.t.	n.t.	n.t.	n.d.
Cinnamyl alcohol	n.t.	n.t.	n.t.	n.t.	n.t.	5.0 ± 0.2
Veratryl alcohol	n.t.	n.t.	n.t.	n.t.	n.t.	5.4 ± 0.2
Vanillyl alcohol	n.t.	n.t.	n.t.	n.t.	n.t.	n.d.

*ABTS coupled assay in 50 mM sodium phosphate buffer, pH 7.0, at room temperature with 300 mM carbohydrate or polyol, or 10 mM aryl alcohol or aldehyde and. 2mg/mL polysaccharide substrates, except for *Nex*GalOx that was assayed in 50mM sodium acetate buffer, pH 5.0. n.d. = Activity not detected at [E] up to 2 mg/mL (27μM). n.t. = Not tested.

Table S3. Sequence identity and similarity of AA5_2 catalytic modules characterized in this work versus previously characterized members.*

	Xpa GalOx	*Bsp* GalOx	*Efe* GalOx	*Nex* GalOx	*Nha* GalOx	*Ast* AAO	***Fgr* GalOx**	***Mre* GalOx**	***Fve* GalOx**	***Fsu* GalOx**	***Fox* AAO**	***Fgr* AAO**	***Fox* AlcOx**	***Cgr* AAO**
*Xpa*GalOx (jgi|Xanpa2|1578647)		57	55	53	54	53	57	59	53	54	53	54	53	46
*Bsp*GalOx (jgi|Bissp1|110239)	71		55	52	52	51	55	56	51	51	51	52	52	46
*Efe*GalOx (ACN30267)	69	68		60	60	58	64	52	57	58	57	58	57	43
*Nex*GalOx (jgi|Nieex1|192818)	69	67	73		62	59	65	53	61	61	61	61	60	47
*Nha*GalOx (XP_003039318)	66	64	69	72		69	71	53	69	69	69	69	70	45
*Ast*AAO (jgi|Acrst|1377707)	67	65	68	72	77		65	51	71	71	71	71	71	45
***Fgr*GalOx (AAO95371)**	69	69	73	77	80	77		55	65	65	64	65	64	47
***Mre*GalOx (XP_033570565)**	72	69	64	66	64	65	66		53	54	53	54	54	46
***Fve*GalOx (ADG08188)**	67	66	70	74	80	81	77	65		97	98	92	82	45
***Fsu*GalOx (ADG08187)**	68	66	70	73	79	80	77	65	98		96	90	81	46
***Fox*AAO (XP_018246910)**	68	65	70	73	79	80	76	65	99	98		91	81	46
***Fgr*AAO (XP_011322138)**	68	66	70	73	79	80	77	65	95	95	95		79	46
***Fox*AlcOx (FOPG_18201)**	67	65	68	71	80	80	76	64	88	88	88	87		46
***Cgr*AAO (EFQ27661)**	63	62	59	62	61	61	62	64	62	62	62	63	62	

*Sequence identity and similarity values are highlighted in green and blue, respectively. Previously characterised AA5_2s are in bold font; see main text for literature references. Percentage coverage of the catalytic module sequences was between 98 – 100%.

Supplementary Figures

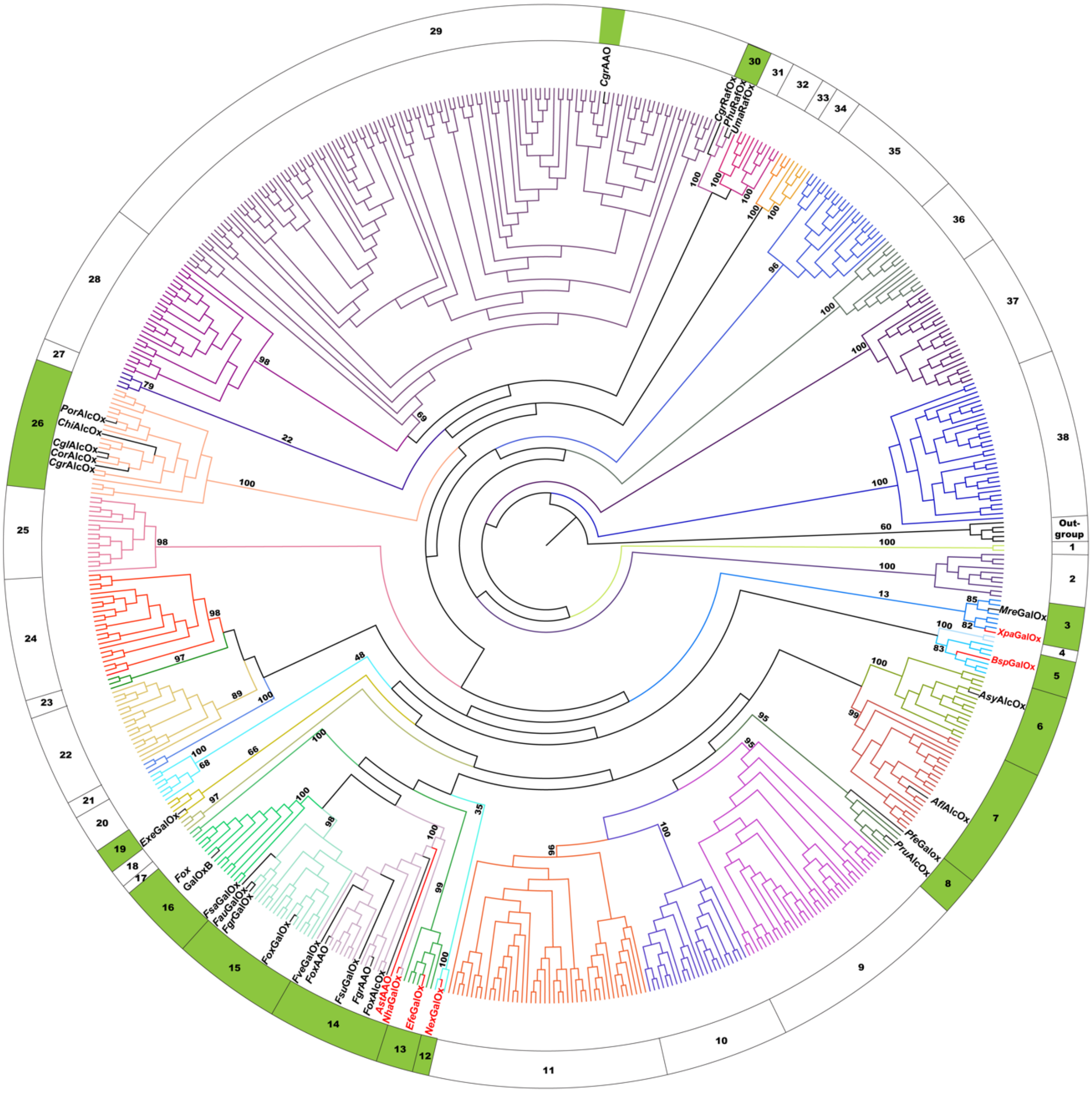

Figure S1. Maximum likelihood phylogeny of AA5_2 members. A maximum likelihood phylogeny was generated with a curated set of 623 AA5_2 catalytic modules omitting any accessory modules, using AA5_1 sequences as an outgroup (1). Each subgroup is distinguished by different branch colors and. numbers. Subgroups containing characterized members are indicated in green. AA5_2 members with previously available biochemical data are labeled in black and. those from this work are in red. Bootstrap values indicated at each node/branch support the 38 subgroups identified.

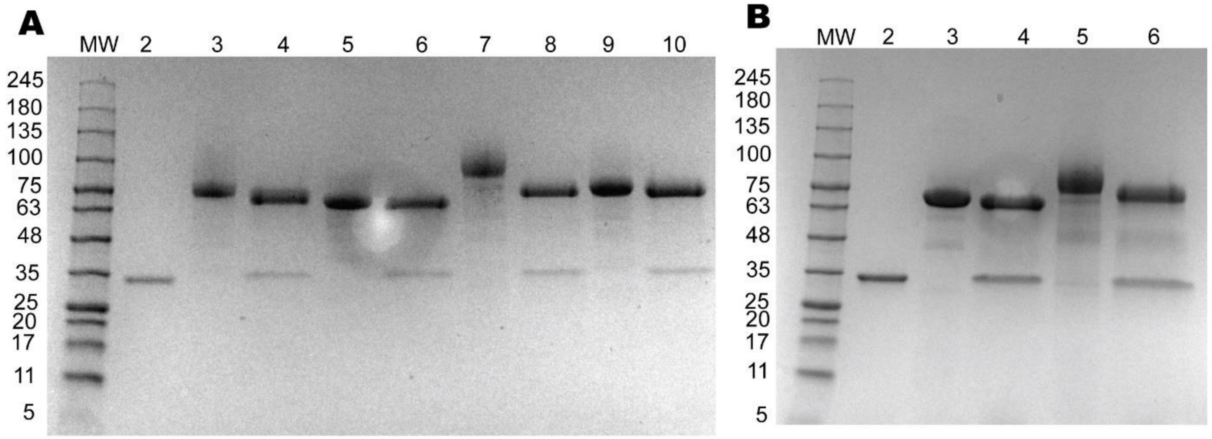

Figure S2. SDS-PAGE of purified AA5_2 members recombinantly produced in *P.pastoris* and N-deglycosylation. Aliquots of enzymes were N-deglycosylated with PNGaseF and 3 µg of protein was loaded. MW = molecular weight markers, kDa. (A) Lane 2: PNGaseF, 3: *Ast*AAO, 4: *Ast*AAO + PNGaseF, 5: *Bsp*GalOx, 6: *Bsp*GalOx + PNGaseF, 7: *Efe*GalOx, 8: *Efe*GalOx + PNGaseF, 9: *Nha*GalOx, 10: *Nha*GalOx + PNGaseF (B) Lane 2: PNGaseF, 3: *Xpa*GalOx, 4: *Xpa*GalOx + PNGaseF, 5: *Nex*GalOx, 6: *Nex*GalOx + PNGaseF. Gels were stained with Coomassie blue.

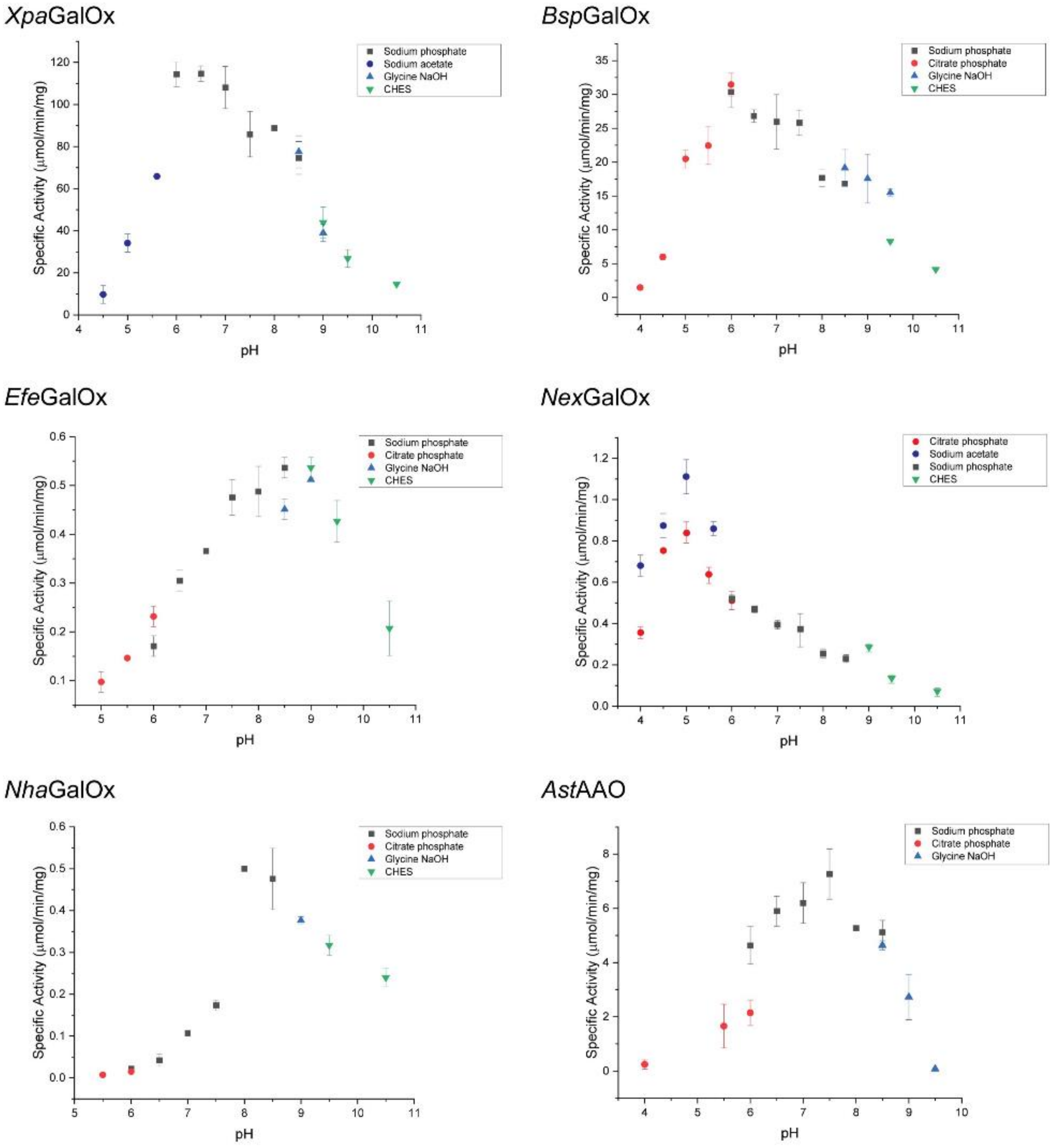

Figure S3. pH-rate profiles. pH-rate profiles were determined using the HRP-ABTS coupled assay with 300mM galactose as the substrate for *Xpa*-,*Bsp*-,*Efe*, *Nex*- and *Nha*-GalOx and 10mM HMF for *Ast*AAO. Measurements were made in triplicate at room temperature. Sodium phosphate (black square), citrate phosphate (red circle), sodium acetate (blue circle), glycine NaOH (blue triangle) and CHES (green triangle) buffers at 50mM were used to cover a pH range of 4-10.5.

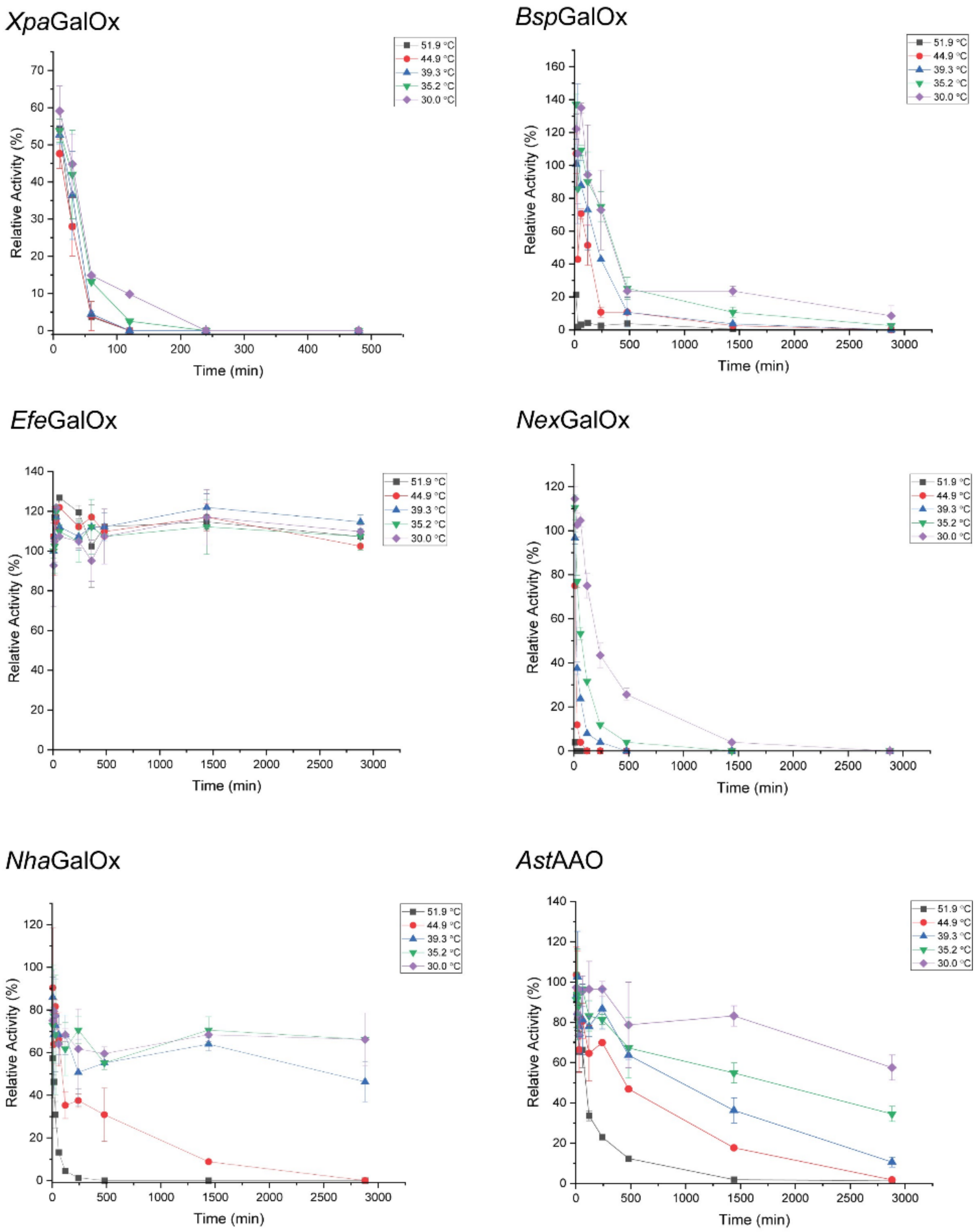

Figure S4. Temperature stability profiles. Activity was determined using the couple HRP-ABTS assay with 300 mM galactose and 10 mM HMF as substrates for the galactose oxidases and alcohol oxidase, respectively. Reactions were performed in duplicate at room temperature and each enzyme was pre-incubated at each temperature and maintained by a gradient thermocycler: 30°C (purple diamond), 35.2°C (green triangle), 39.3°C (blue triangle), 44.9°C (red circle) and 51.9°C (black square).

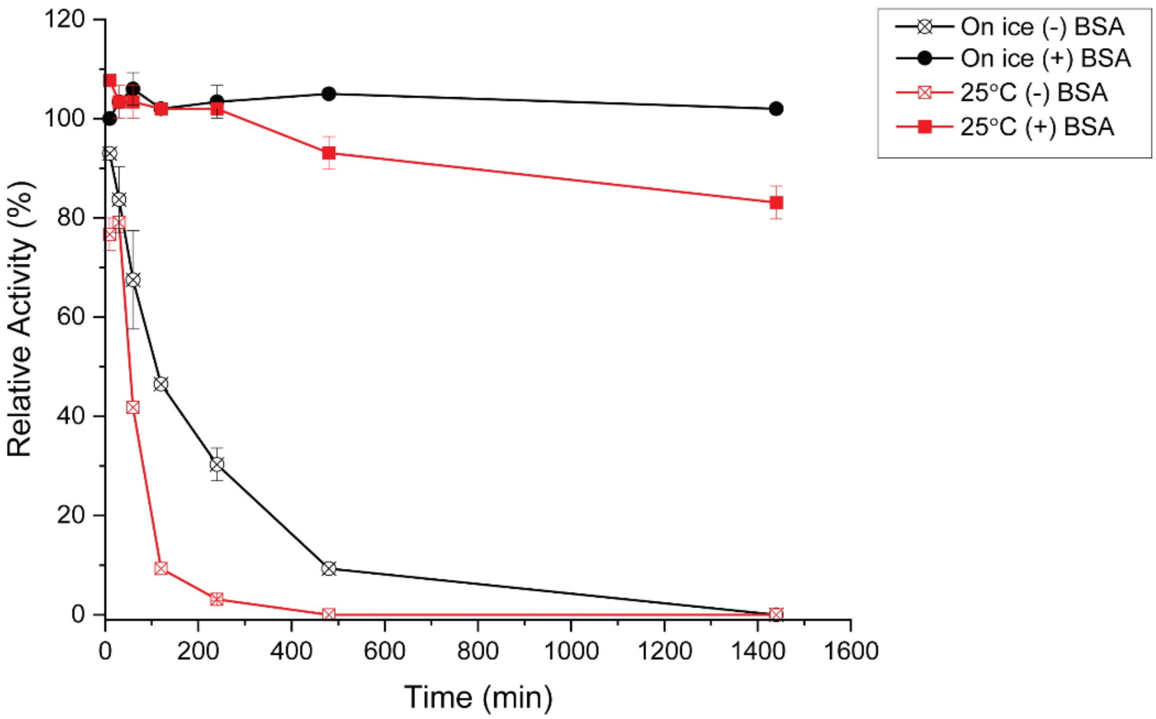

Figure S5. Effects of BSA addition on observed activity of *Xpa*GalOx. Due to the high activity of *Xpa*GalOx, diluted solutions of enzyme required stabilisation by BSA. A working concentration of 5×10^4 mg mL^{-1} (7.3 nM) was used.

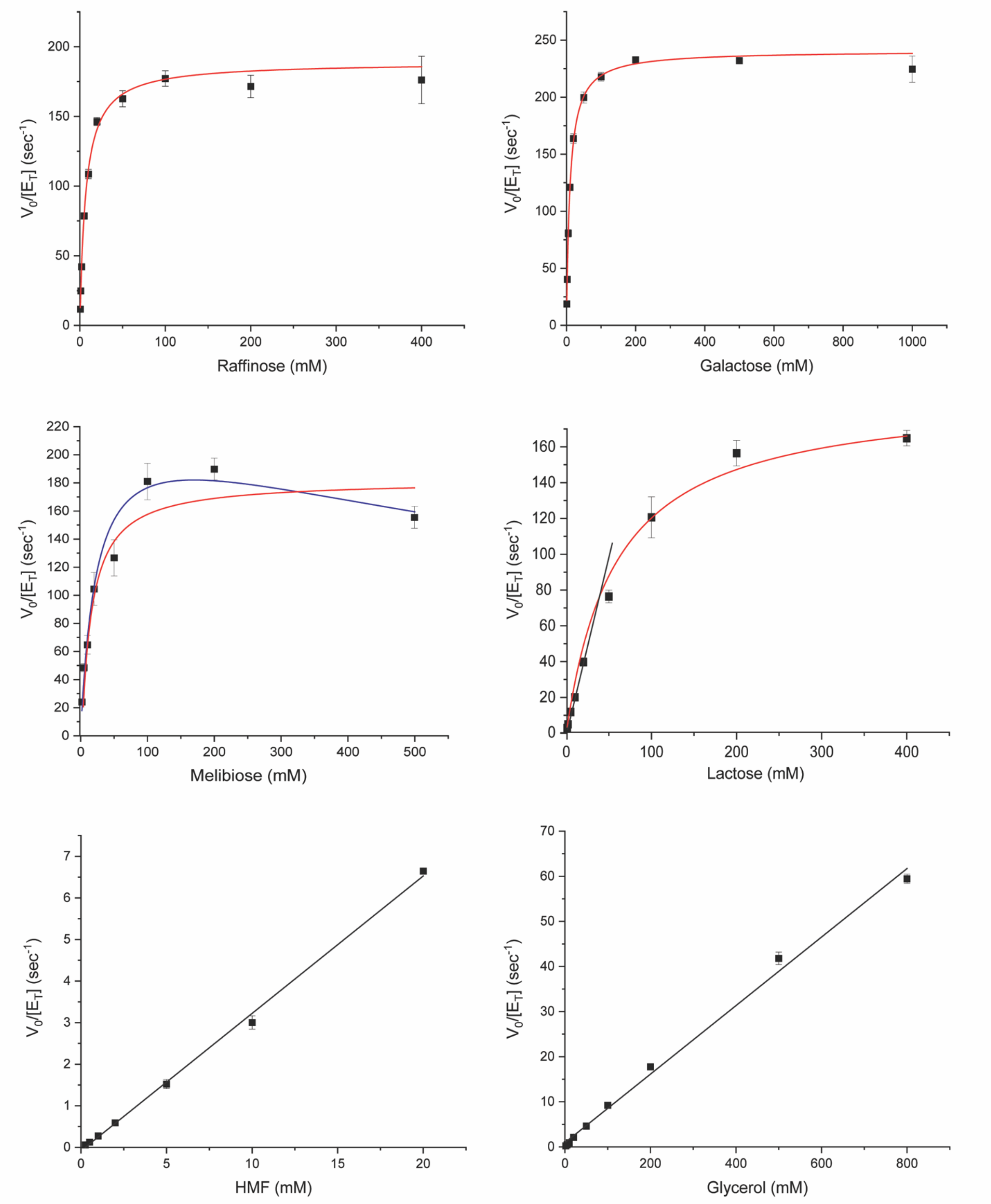

Figure S6. *Xpa*GalOx Michaelis-Menten kinetics. Initial rates were measured in triplicate at each substrate concentration. The individual k_{cat} and K_M values were calculated by performing a non-linear fitting analysis of the standard Michaelis-Menten equation (red line) using OriginLab 9.85. A linear fit (black line) was applied to data from reactions that did not reach saturation. *Xpa*GalOx displayed substrate inhibition (blue line) when assayed on melibiose.

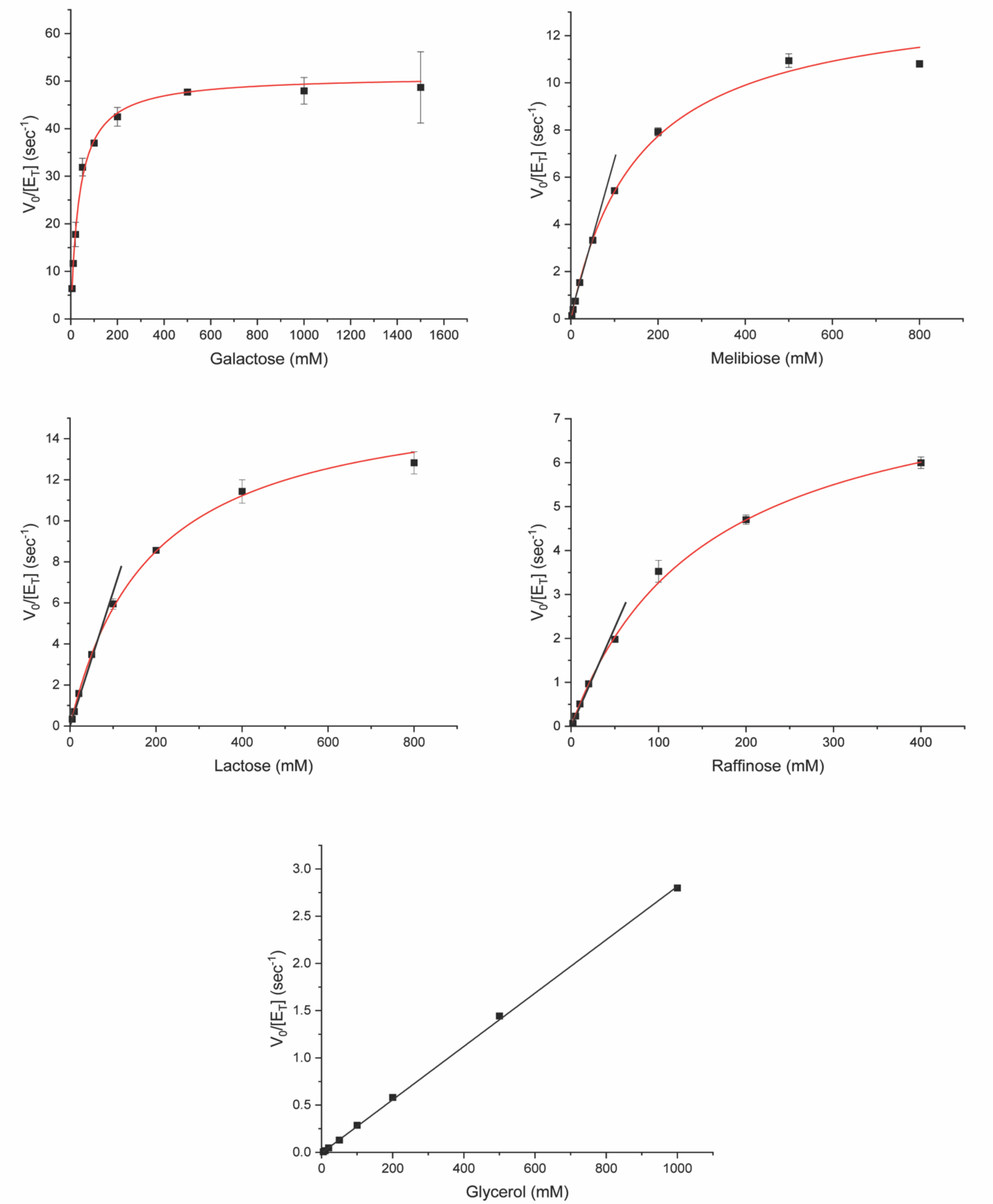

Figure S7. *Bsp*GalOx Michaelis-Menten kinetics. Initial rate values were measured in triplicate at each substrate concentration. The individual k_{cat} and K_M values were calculated by performing a non-linear fitting analysis of the standard Michaelis-Menten equation (red line) using OriginLab 9.85. A linear fit (black line) was also applied to the data from reactions where saturation kinetics were not observed.

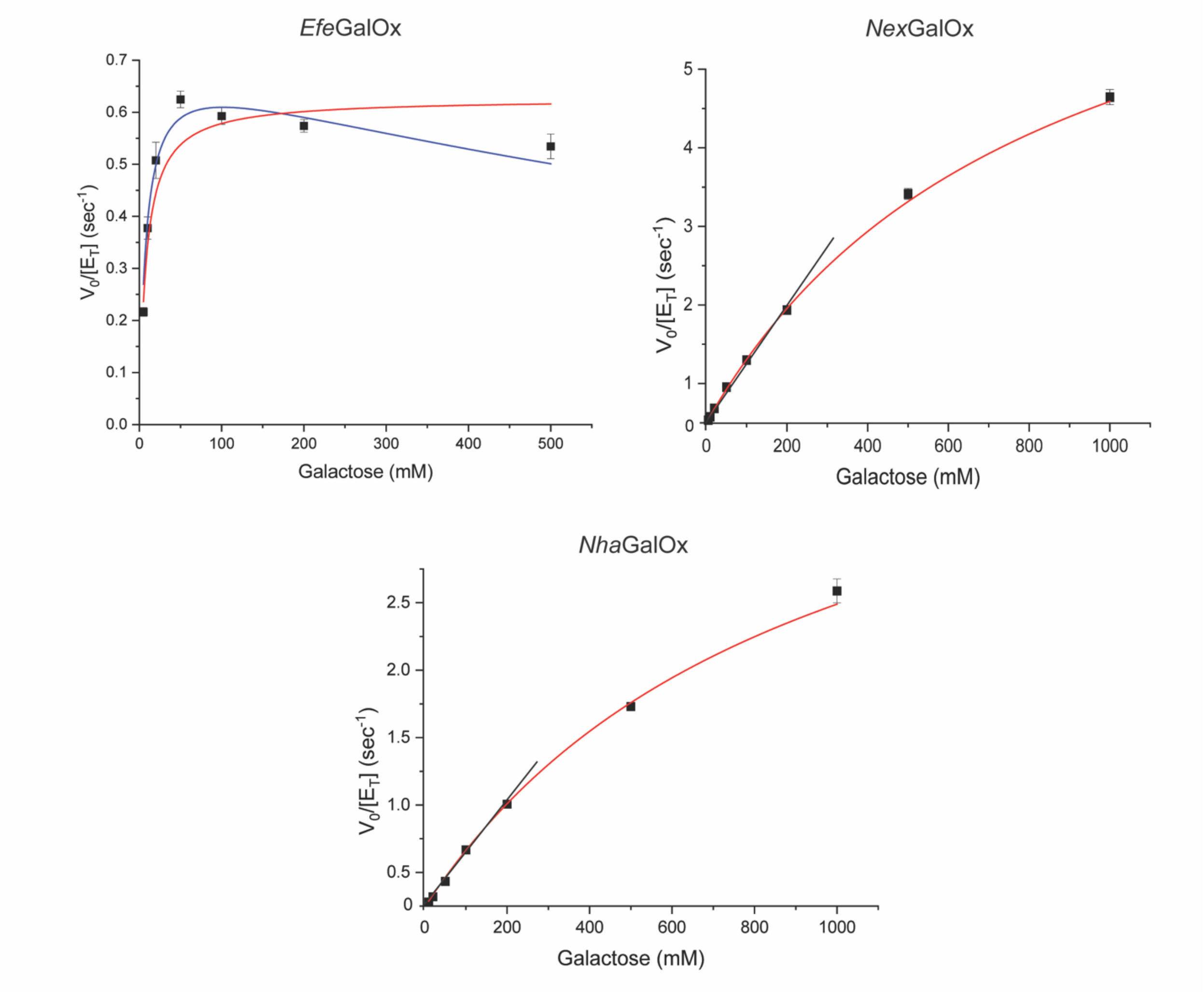

Figure S8. *Efe*GalOx, *NexGalOx* and *Nha*GalOx Michaelis-Menten kinetics. Initial rate values were measured in triplicate at each substrate concentration. The individual k_{cat} and K_M values were calculated by performing a non-linear fitting analysis of the standard Michaelis-Menten equation (red line) using OriginLab 9.85. A linear fit (black line) was also applied to data from reactions that did not reach saturation. *Efe*GalOx displayed substrate inhibition (blue line) when assayed on galactose.

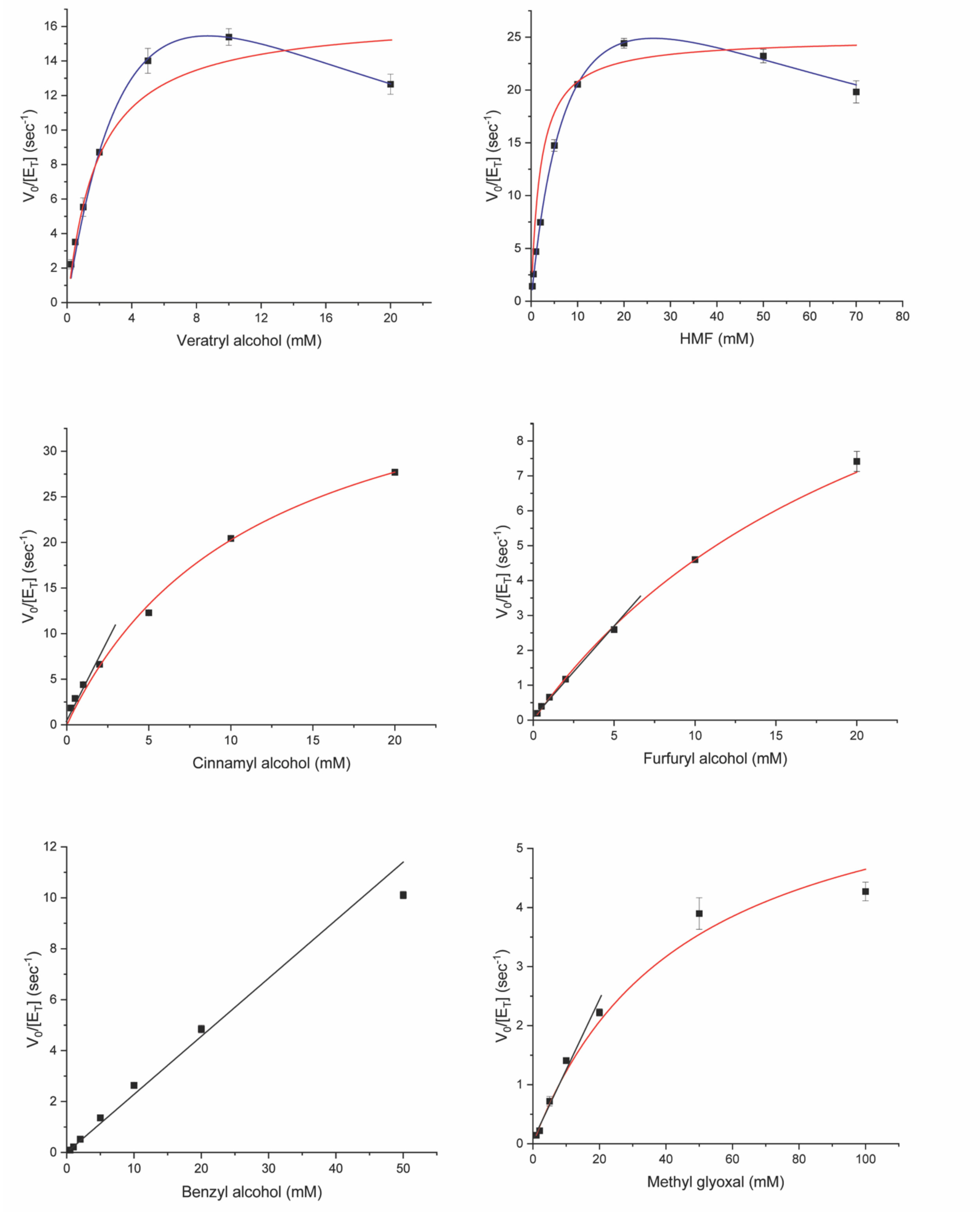

(continued on next page)

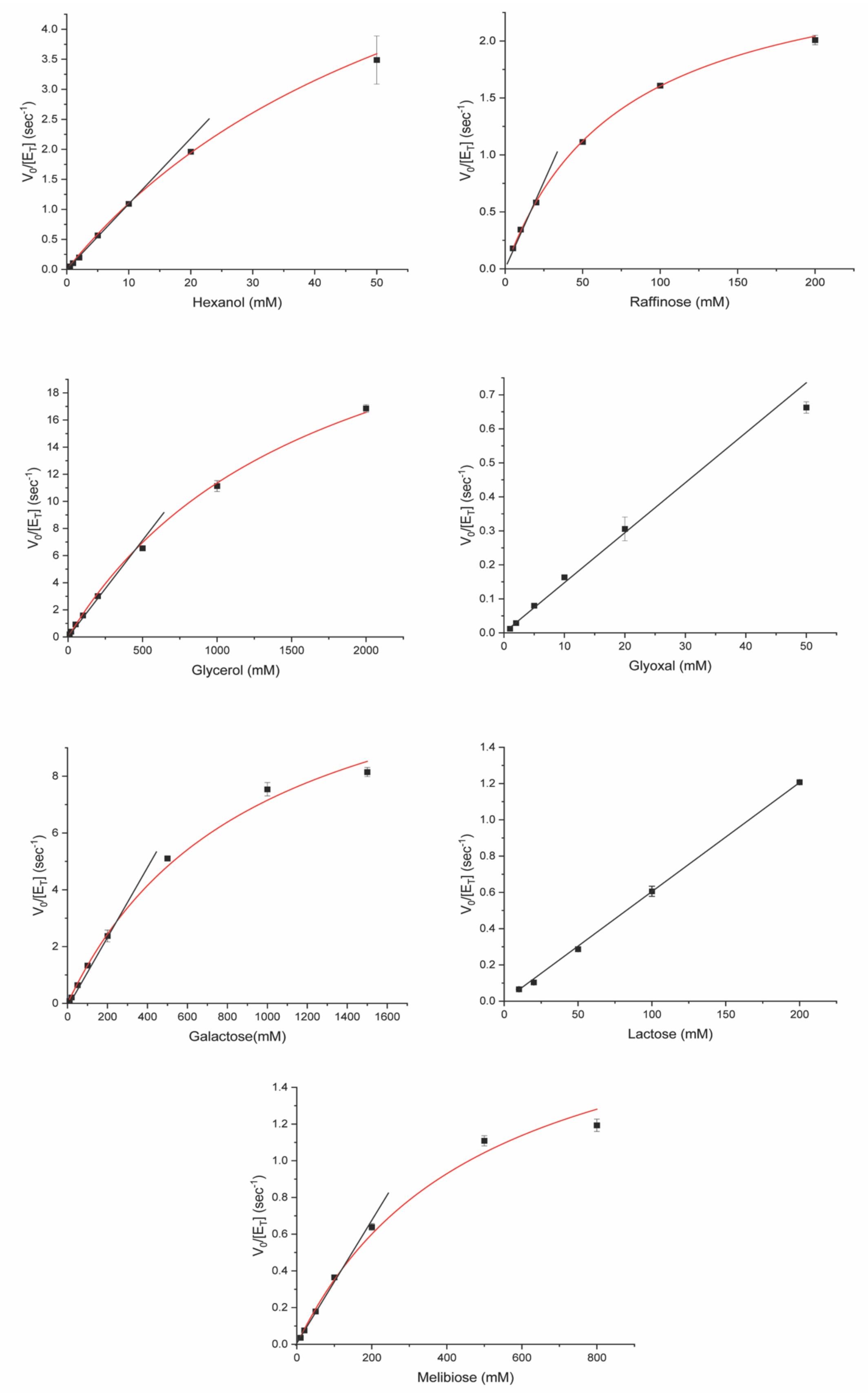
V0/[ET] (sec-1)
Hexanol (mM)
Raffinose (mM)
Glycerol (mM)
Glyoxal (mM)
Galactose(mM)
Lactose (mM)
Melibiose (mM)

Figure S9. *Ast*AAO Michaelis-Menten kinetics. Initial rate values were measured in triplicate at each substrate concentration. The individual k_{cat} and K_M values were calculated by performing a non-linear fitting analysis of the standard Michaelis-Menten equation (red line) using OriginLab 9.85. A linear fit (black line) was also applied to data from reactions that did not reach saturation. *Ast*AAO displayed substrate inhibition (blue line) when assayed on veratryl alcohol and HMF.

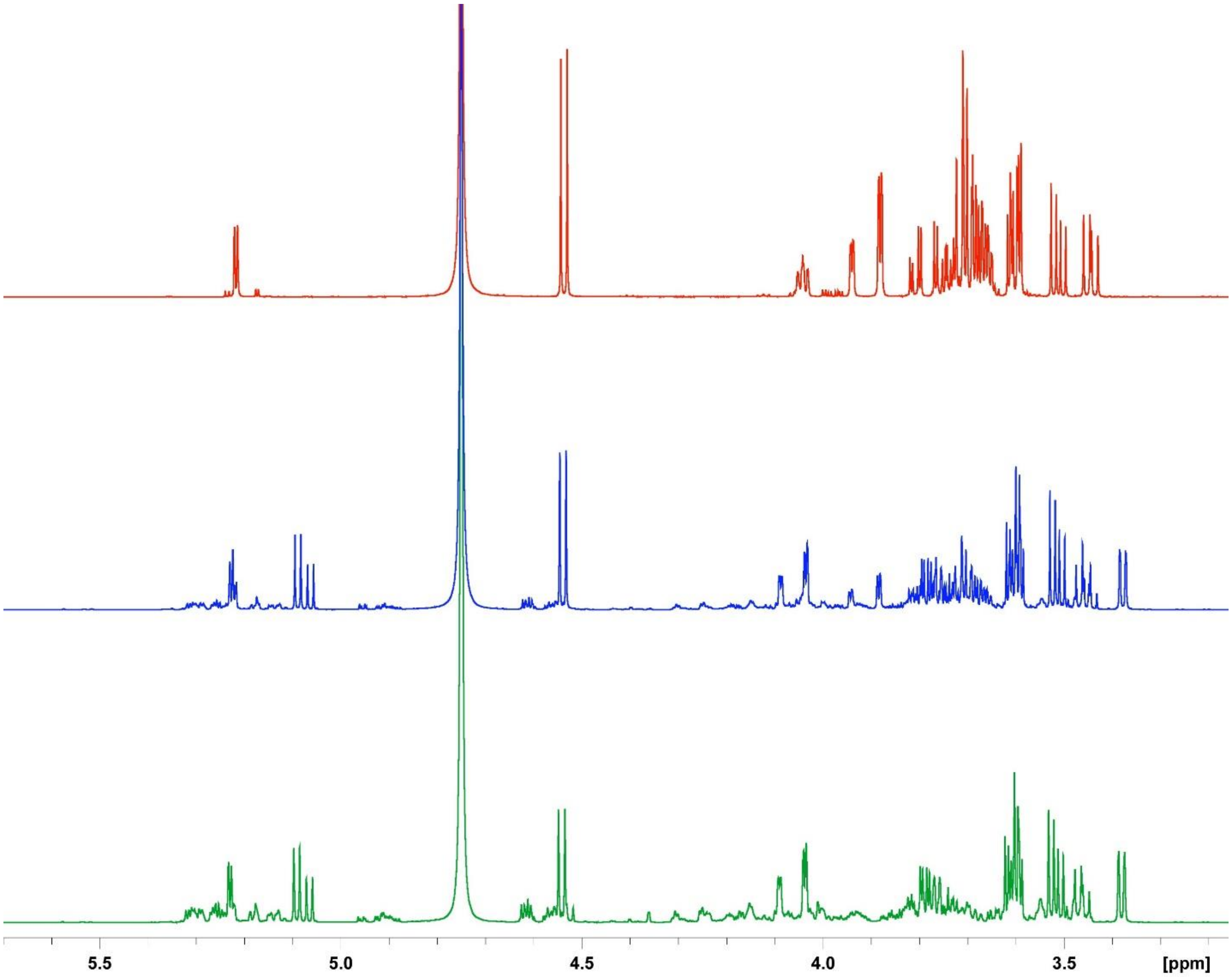

Figure S10. Galactose oxidation by *Xpa*GalOx. ^{1}H NMR spectra for the negative control reaction (red), reaction with *Xpa*GalOx (blue) and reaction with *Xpa*GalOx and 0.1 mg/mL BSA added (green). A 1:1 ratio of HRP: catalase was added and reactions were carried out for 24 hours at ambient temperature with stirring at 400 rpm. A concentration of 0.7 μM (50μg) *Xpa*GalOx was used in a 1 mL reaction.

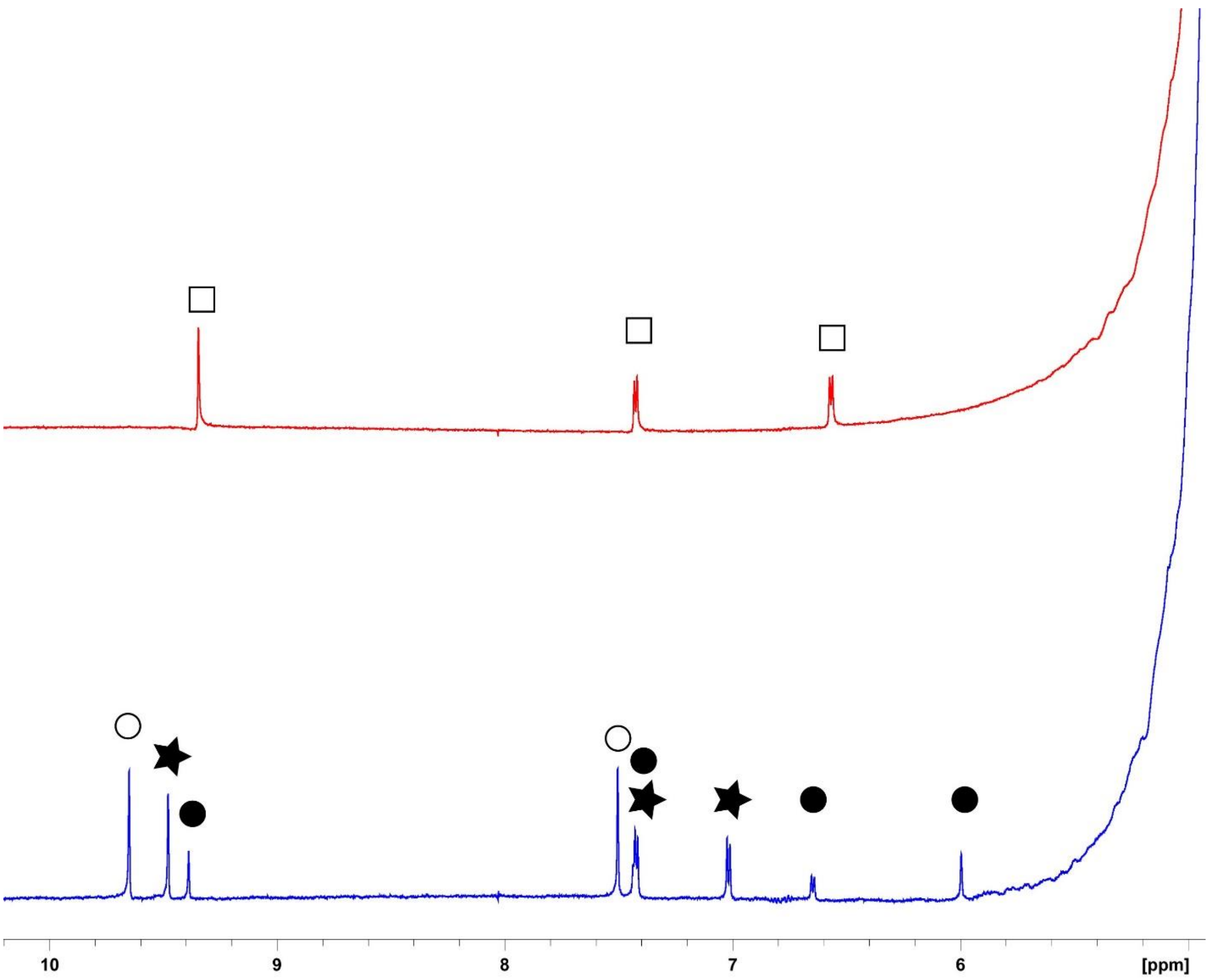

Figure S11. HMF oxidation by *Ast*AAO. ^{1}H NMR spectra for the negative control reaction (red) and reaction with *Ast*AAO (blue). A 1:1 ratio of HRP: catalase was added, and reactions were carried out for 24 hours at ambient temperature with stirring at 400 rpm. A concentration of 1.4 μM (100 μg) *Ast*AAO was used in a 1 mL reaction. HMF, DFF, DFF hydrate and FFCA are denoted by open squares, open circles, black circles and black stars, respectively. Peaks were assigned versus reference spectra (1, 2) .

Supplementary References

1. **Cleveland ME, Mathieu Y, Ribeaucourt D, Haon M, Mulyk P, Hein JE, Lafond M, Berrin JG, Brumer H.** 2021. A survey of substrate specificity among Auxiliary Activity Family 5 copper radical oxidases. Cellular and Molecular Life Sciences **78:**8187-8208.
2. **Carro J, Ferreira P, Rodriguez L, Prieto A, Serrano A, Balcells B, Arda A, Jimenez-Barbero J, Gutierrez A, Ullrich R, Hofrichter M, Martinez AT.** 2015. 5-hydroxymethylfurfural conversion by fungal aryl-alcohol oxidase and unspecific peroxygenase. Febs Journal **282:**3218-3229.
